# Intrinsic nucleus-targeted ultra-small metal–organic framework for the type I sonodynamic treatment of orthotopic pancreatic carcinoma

**DOI:** 10.1186/s12951-021-01060-7

**Published:** 2021-10-12

**Authors:** Tao Zhang, Yu Sun, Jing Cao, Jiali Luo, Jing Wang, Zhenqi Jiang, Pintong Huang

**Affiliations:** 1 Department of Ultrasound in Medicine, The Second Affiliated Hospital of Zhejiang University School of Medicine, No.88 Jiefang Road, Shangcheng District,, Hangzhou, 310009 People’s Republic of China; 2 Research Center of Ultrasound in Medicine and Biomedical Engineering, The Second Affiliated Hospital of Zhejiang University School of Medicine, No.88 Jiefang Road, Shangcheng District, Hangzhou, 310009 People’s Republic of China; 3 Institute of Engineering Medicine, Beijing Institute of Technology, No. 5, South Street, Zhongguancun, Haidian District, Beijing, 100081 People’s Republic of China

**Keywords:** Type I sonodynamic therapy, Intrinsic nucleus-targeted, Hypoxia, Ultra-small metal–organic framework, Orthotopic pancreatic carcinoma

## Abstract

**Background:**

Sonodynamic therapy (SDT) strategies exhibit a high tissue penetration depth and can achieve therapeutic efficacy by facilitating the intertumoral release of reactive oxygen species (ROS) with a short lifespan and limited diffusion capabilities. The majority of SDT systems developed to date are of the highly O_2_-dependent type II variety, limiting their therapeutic utility in pancreatic cancer and other hypoxic solid tumor types.

**Results:**

Herein, a nucleus-targeted ultra-small Ti-tetrakis(4-carboxyphenyl)porphyrin (TCPP) metal–organic framework (MOF) platform was synthesized and shown to be an effective mediator of SDT. This MOF was capable of generating large quantities of ROS in an oxygen-independent manner in response to low-intensity ultrasound (US) irradiation (0.5 W cm^−2^), thereby facilitating both type I and type II SDT. This approach thus holds great promise for the treatment of highly hypoxic orthotopic pancreatic carcinoma solid tumors. This Ti-TCPP MOF was able to induce in vitro cellular apoptosis by directly destroying DNA and inducing S phase cell cycle arrest following US irradiation. The prolonged circulation, high intratumoral accumulation, and nucleus-targeting attributes of these MOF preparations significantly also served to significantly inhibit orthotopic pancreatic tumor growth and prolong the survival of tumor-bearing mice following Ti-TCPP + US treatment. Moreover, this Ti-TCPP MOF was almost completely cleared from mice within 7 days of treatment, and no apparent treatment-associated toxicity was observed.

**Conclusion:**

The nucleus-targeted ultra-small Ti-TCPP MOF developed herein represents an effective approach to the enhanced SDT treatment of tumors in response to low-intensity US irradiation.

**Graphic abstract:**

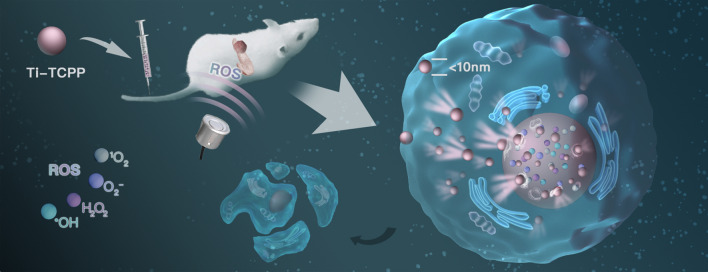

**Supplementary Information:**

The online version contains supplementary material available at 10.1186/s12951-021-01060-7.

## Background

Sonodynamic therapy (SDT) is a relatively non-invasive approach to treating a range of cancer types [1, 2]. SDT combines the advantage of a high tissue penetration depth with the ability to induce the generation of reactive oxygen species (ROS) in order to kill tumor cells [3–8]. While promising, most SDT approaches rely on an O_2_-dependent type II SDT modality, limiting their utility in solid tumors [9–11]. In contrast, type I SDT is more hypoxia-tolerant as it relies upon the generation of cytotoxic radicals and superoxide anions, which can better kill tumors under hypoxic conditions. Pancreatic tumors are often considered to be the most hypoxic of all tumor types on average, with an average O_2_ pressure of less than 2.5 mmHg in up to 0–16% of tumor area as compared to 30–50 mmHg in normal tissues [12–14]. Improving the utility of SDT in such hypoxic tumors is thus dependent upon the development of effective type I SDT strategies that can generate ROS under low O_2_ conditions [15, 16].

Another key determinant of SDT efficacy is the subcellular localization of sonosensitizing agents [17–19], as most generated ROS exhibit a very brief lifespan (< 40 ns) and a limited diffusion length (~ 20 nm) [20, 21]. Recent evidence suggests that nanoagents located closer to DNA are better able to induce oxidative damage and to thereby achieve superior therapeutic efficacy [22–24]. This has led to efforts to target nanoparticles to cellular nuclei through approaches such as targeted design strategies and the utilization of particles with a positive surface charge [25]. Such targeting approaches typically rely on the modification of the type or density of surface ligands including peptides or adenoviral vectors so as to better target these particles to particular receptors that are expressed in cells of interest [21, 26, 27]. However, targeted aggregation within the nucleus can be limited by several factors, with the size of the nanoparticle being the most commonly studied of these limitations. It has been reported that nanoparticles smaller than 50 nm in diameter can be delivered to the nucleus in a targeted manner [26]. Moreover, although cationic nanoparticles can better accumulate in the cell nucleus [28–30], they are also easily nonspecifically absorbed by other cells and can be quickly cleared from circulation owing to their positively charged nature.

Nanoscale metal–organic frameworks (MOFs) are composed of self-assembling metal ions and organic ligands and have been widely used in the context of tumor treatment owing to their porosity and other valuable structural and chemical properties [31–36]. We have previously reported the development of a nucleus-targeted MOF structure with a high photothermal conversion rate in response to strong near-infrared (NIR) light absorbance that was able to facilitate efficient tumor treatment [37]. Similar MOFs that are targeted to the nuclei and that can efficiently generate ROS may be ideal therapeutic agents to facilitate SDT tumor treatment. However, there have been relatively few reports of sonosensitizers that efficiently function in response to US irradiation [38, 39], and even fewer exhibit intrinsic nucleus-targeted activity and good biodegradability in vivo [11]. Ultra-small MOFs are considered to be highly dispersible [40] and can be efficiently metabolized in vivo in biomedical contexts [41]. These MOFs can also be utilized for intrinsic nuclear targeting owning to their ultra-small size characteristics, yet there have been few reports to date exploring this approach.

To overcome these limitations, it is thus important that a sonostable, biocompatible sonosensitizer capable of targeting to nuclei of tumor cells and generating ROS therein in an oxygen-independent manner be developed.

**Scheme 1.**
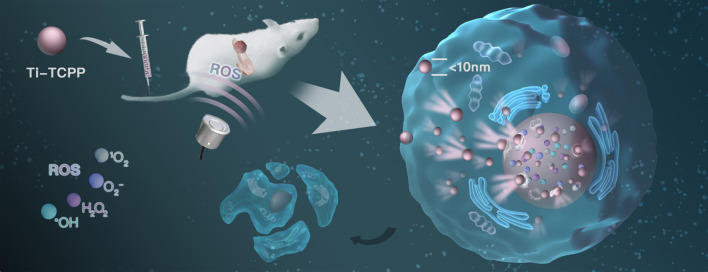
A schematic illustration of ultra-small Ti-TCPP MOF application in nuclear-targeted SDT

Herein, we report the development of an intrinsic nuclear-targeted Ti-tetrakis(4-carboxyphenyl)porphyrin (TCPP) MOF that was utilized as a sonosensitizer in an effort to overcome the limited efficacy of SDT for the treatment of orthotopic pancreatic carcinoma (Scheme 1). This novel MOF was able to be effectively internalized into cells owing to its small size (< 10 nm) and its charge reversal property [42, 43], enabling it to be directly targeted to the nuclei and to thereby facilitate more efficient SDT. Moreover, this Ti-TCPP MOF platform was confirmed to generate ROS in a hypoxic environment, thereby facilitating oxygen-independent SDT treatment. Furthermore, our Ti-TCPP MOF exhibited good biodegradability and safety in vitro and in vivo. As such, we believe that this ultra-small Ti-TCPP MOF holds great promise for the treatment of hypoxic tumor types including pancreatic cancer.

## Experimental

### Ti-TCPP MOF synthesis

TiCl_4_·2THF (4 mg) was dissolved in 1 mL N,N-dimethylformamide (DMF) and combined with 5,10,15,20-tetra(p-benzoato)porphyrin (H^4^TBP) (2 mg) that had been dissolved in 2 mL of DMF. Next, this solution was combined with 200 μL of acetic acid (AcOH). This solution was then mixed and incubated for 4 h at 90 °C. Samples were then washed, spun down, and 10 mg of the resultant products were combined with 5 mL of dimethyl sulfoxide (DMSO) and sonicated for 24 h in a horn sonicator (Branson Digital Sonifier SFX 550, Carouge, Switzerland) at 150 W.

### ROS generation

Singlet oxygen (^1^O_2_) generation was assessed with a singlet oxygen sensor green (SOSG) probe (Thermo Fisher Scientific, MA, USA) (ex/em: 504/525 nm). Superoxide (O_2_^−^) generation was assessed using dihydrorhodamine 123 (DHR 123, Sigma-Aldrich, USA) (ex/em: 488/535 nm). Hydrogen peroxide (H_2_O_2_) generation was detected at the wavelength of 560 nm with a hydrogen peroxide assay kit (S0038, Beyotime, China). Hydroxyl radical (·OH) generation was measured via aminophenyl fluorescein (APF) assay (Sigma-Aldrich, USA) (ex/em: 490/515 nm). Briefly, Ti-TCPP MOF was suspended in PBS at equivalent Ti concentrations of 0, 10, 20, 40, 80, and 160 µg mL^−1^. These solutions were then exposed to US irradiation (0.5 W cm^−2^, duty rate 50%, 1 min, 1 MHz, Mettler Sonicator 740), after which fluorescence was analyzed with a multiscan spectrum (Tecan, Swiss).

### Assessment of Ti-TCPP MOF cellular uptake

BxPC-3 cells were plated in 6-well 0.01% poly (Lys)-coated plates (1 × 10^5^ cells well^−1^) overnight, after which fresh media containing 10 μg mL^−1^ of Ti-TCPP MOF was added for 1, 2, 6, or 8 h. Cells were then washed three times, collected, and analyzed with a FACSCalibur flow cytometer (BD, USA).

Laser scanning confocal microscopy (LSCM) was additionally used to assess Ti-TCPP MOF uptake. For these experiments, BxPC-3 cells (1 × 10^5^) were grown overnight in 2 mL in confocal culture dishes (NETS Co., USA), after which 10 μg mL^−1^ of Ti-TCPP MOF or PBS were added for 1, 2, or 6 h. cells were then washed three times with PBS, fixed for 30 min with 4% formaldehyde, and stained with Hoechst 33258 Stain solution (10 μg mL^−1^) for 30 min prior to LSCM assessment.

Additionally, nuclear Ti levels were assessed via inductively coupled plasma optical emission spectrometry (ICP-OES) with an Optima 2100DV instrument (Perkin Elmer, USA). Mass was calculated on a per-cell basis. Briefly, BxPC-3 cells were incubated overnight and then treated by Ti-TCPP MOF (5 μg/mL for Ti) for 1, 2, 6 and 8 h. After washing with PBS for three times, cells were collected, and nuclei were extracted via nucleus extraction.

### Cell viability assay

Ti-TCPP MOF biocompatibility was assessed via a Cell Counting Kit-8 (CCK-8) assay (MCE, USA). Briefly, BxPC-3, Panc02, or hTERT-HPNE cells were added to 96-well plates (5000 cells well^−1^) overnight, after which media was exchanged for DMEM/1640 containing a range of Ti-TCPP MOF concentrations. Following a 24 h incubation, CCK-8 solution was added to each well and a microplate reader was used to assess absorbance at 450 nm. The efficiency of SDT in vitro was also assessed by adding 100 µL of BxPC-3 cells to individual wells of 96-well plates overnight, after which media containing a range of Ti-TCPP MOF concentrations was added for 6 h. Cells were then subjected to low-intensity US treatment for 1 min (0.5 W cm^−2^, 1 MHz, 50% duty cycle). A CCK-8 assay was then used to assess viability as above. In other experiments, BxPC-3 cells were added to 6-well plates and grown in the presence of 10 µg mL^−1^ Ti-TCPP MOF for 6 h. Following US irradiation for 1 min (0.5 W cm^−2^, 1 MHz, 50% duty cycle) and another 18 h incubation, cells were stained using PI and/or Annexin V-FITC, after which they were assessed via flow cytometry.

### In vitro DNA damage analysis

The immunofluorescence staining of BxPC-3 cells was performed to detect DSBs. Following appropriate treatments, cells were washed and fixed in 4% paraformaldehyde for 15 min. Cells were then stained with rabbit monoclonal anti-H2AX (1:1500) overnight at 4 °C, after which they were incubated with AlexaFluor 488-conjugated anti-rabbit secondary antibody (1:400) and 2.0 mg mL^−1^ DAPI for 30 min. Cells were then imaged with a Leica fluorescence microscope (200×).

In the DNA Ladder assay, appropriately treated BxPC-3 cells were lysed for 0.5 h, and supernatants were collected after centrifugation. A DNA Ladder kit (Beyotime Institute of Biotechnology, China) was then used based upon provided instructions to separate DNA, which was run on a 0.8% agarose gel.

### Orthotopic tumor model

Female nude mice (5–6 weeks old, BiKai Biological, Nanjing, China) were used for all animal studies, which were approved by the Regional Ethics Committee for Animal Experiments at The Second Affiliated Hospital of Zhejiang University School of Medicine (Permit No. 2019-070). Mice were anesthetized and a 1 cm incision in the upper left abdominal quadrant was made. The spleen and tail of the pancreas were then exposed, and 50 μL of BxPC-3 cells labeled with firefly luciferase suspended in PBS and Matrigel (phenol red-free, 2:3) were injected into the tail of the pancreas using a 0.3 mm needle. The spleen and pancreas were then restored to their appropriate positions within the abdomen, and the peritoneum was sutured using 4–0 absorbable sutures, after which the skin was closed with 6–0 non-absorbable sutures. Animals were then placed on a warming blanket until fully recovered from anesthetization.

### Pharmacokinetics and bio-distribution

The pharmacokinetics of Ti-TCPP MOF assessed by injecting 100 μL Ti-TCPP MOF (10.0 mg kg^−1^) into the mice through the tail vein. Blood samples were then collected at different time points (0.17, 0.5, 1, 2, 4, 8, 12, 18 and 24 h), lyophilized, weighed and digested with aqua regia. The Ti content in the blood was then analyzed by ICP-OES.

To evaluate the distribution of Ti-TCPP MOF in vivo, tumor-bearing mice were injected 100 µL of a Ti-TCPP MOF solution (10.0 mg kg^−1^) or PBS (pH 7.4). Ti clearance in vivo was assessed by injecting six tumor-bearing mice with 100 µL of Ti-TCPP MOF (10.0 mg kg^−1^). Three mice were then euthanized at baseline and three were euthanized at 8 h post-injection, at which time major organs and tumors were collected and Ti levels were assessed via ICP-OES analysis.

### In vivo fluorescence/PA imaging and therapy

Mice were monitored until tumors had grown to 40–60 mm^3^ in size. After mice were injected 100 µL of a Ti-TCPP MOF solution (10.0 mg kg^−1^) or PBS (pH 7.4), fluorescence and PA imaging (performed at 710 nm) were then conducted at 0, 2, 4, 8, and 12 h post-injection. Tumor-bearing mice were randomly assigned to four treatment groups (five per group): PBS, PBS + US, Ti-TCPP MOF, and Ti-TCPP MOF + US groups. Mice in the indicated treatment groups were injected with 100 µL of PBS (pH 7.4) or 100 µL PBS (pH 7.4) containing Ti-TCPP MOF (10.0 mg kg^−1^). At 8 h post-injection, US irradiation was conducted in the indicated treatment groups (5 min, 0.5 W cm^−2^, 1 MHz, 50% duty cycle). Then the treatment process was repeated every three days, with three treatments in total. After the intraperitoneal injection of 4 mg of d-luciferin in 200 µL of PBS, tumor sizes were assessed within 30 min using an IVIS spectrum pre-clinical in vivo imaging system. Murine survival and tumor growth were monitored for 60 days, after which major organs were collected and stained with hematoxylin and eosin (H&E), Ki67, γ-H2AX, or tdT-mediated dUTP nick-end labeling (TUNEL) and assess via optical microscopy (DMI3000, Leica, Germany).

### Statistical analysis

All experimental results were based on data from at least three independent measurements (n ≥ 3), and all data are presented as means ± standard deviation (SD). Graphpad Prism (version 9.0, GraphPad Software Inc.) was used for all statistical comparisons. Data were analyzed via Student’s *t*-test. *P < 0.05, **P < 0.01, ***P < 0.001.

## Results and discussion

### Ti-TCPP MOF preparation and characterization

**Fig. 1:**
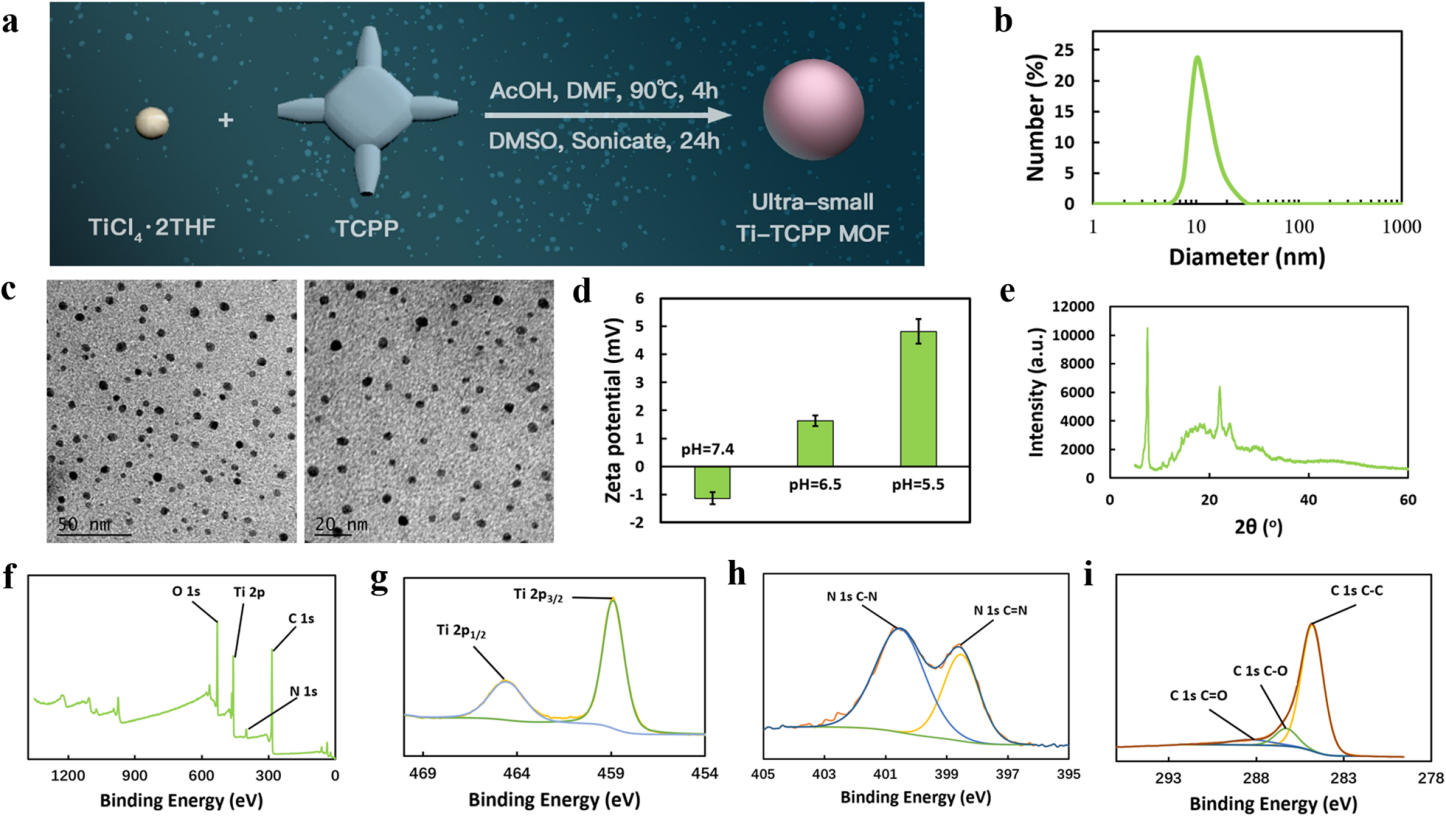
**a** The method of ultra-small Ti-TCPP MOF synthesis. DLS curves (**b**), TEM images (**c**), zeta potentials (**d**), XRD pattern (**e**) and XPS spectra of Ti-TCPP MOF (**f**–**i**)

The approach to the preparation of our sonosensitizer (Ti-TCPP) is shown in Fig. 1a and Additional file 1: Fig. S1. Dynamic light scattering (DLS) indicated that the resultant Ti-TCPP MOF had an average diameter of 12.21 ± 1.27 nm with a polydispersity index of 0.17 (Fig. 1b), and transmission electron microscopy (TEM) (Fig. 1c) revealed uniform monodispersed round Ti-TCPP MOF particles with an average diameter of 5.85 nm. Ti-TCPP MOF particles were able to remain relatively stable for 21 days in PBS and 7 days in FBS at 4 °C (Additional file 1: Fig. S2 and S3), and for 3 days in PBS and cell culture medium at 37 °C without any significant shifts in particle diameter (Additional file 1: Fig. S4). Under US irradiation (0.5 W cm^−2^, 1 MHz, 50% duty cycle, 1 min), the Ti-TCPP MOF was stable, but it did exhibit an increase in size, which may be related to the catalytic reaction caused by US irradiation (Additional file 1: Fig. S5). These Ti-TCPP MOF preparations exhibited a change in zeta potential from − 1.136 to 4.821 mV upon the introduction of excess surface carboxyl groups under different pH conditions (Fig. 1d). Powder X-ray diffraction (XRD) measurements additionally confirmed successful Ti-TCPP MOF synthesis (Fig. 1e), the detailed crystal structure was similar to that previously published by Lan et al. [44]. XPS was additionally used to assess the elemental composition and chemical state of Ti-TCPP MOF preparations, revealing that samples contained C, O, N, and Ti (Fig. 1f). The Ti 2p XPS spectra for these preparations exhibited two peaks at 464.58 and 458.88 eV that were assigned to the emission from Ti 2p_1/2_ and Ti 2p_3/2_, respectively (Fig. 1g). Figure 1h demonstrated the high-resolution N 1 s spectrum of the Ti-TCPP MOF, with two peaks at 400.58 and 398.58 eV corresponding to C–N and C=N, while Fig. 1i showed the high-resolution C 1 s spectrum for this sample. Three characteristic peaks at 288.38, 286.28, and 284.78 eV are attributable to C=O, C–O, and C–C, respectively, indicating that the C compound in this sample was produced using TCPP. We also detected the Brunauer–Emmett–Teller (BET) N_2_ adsorption/desorption of Ti-TCPP MOF (Additional file 1: Fig. S6). The N_2_ adsorption/desorption curve revealed type IV sorption with a surface area of 562.47 m^2^ g^−1^. These data confirmed that we had successfully synthesized an ultra-small Ti-TCPP MOF.

### Assessment of the in vitro ROS-generating efficacy and PA imaging properties of Ti-TCPP MOF

**Fig. 2:**
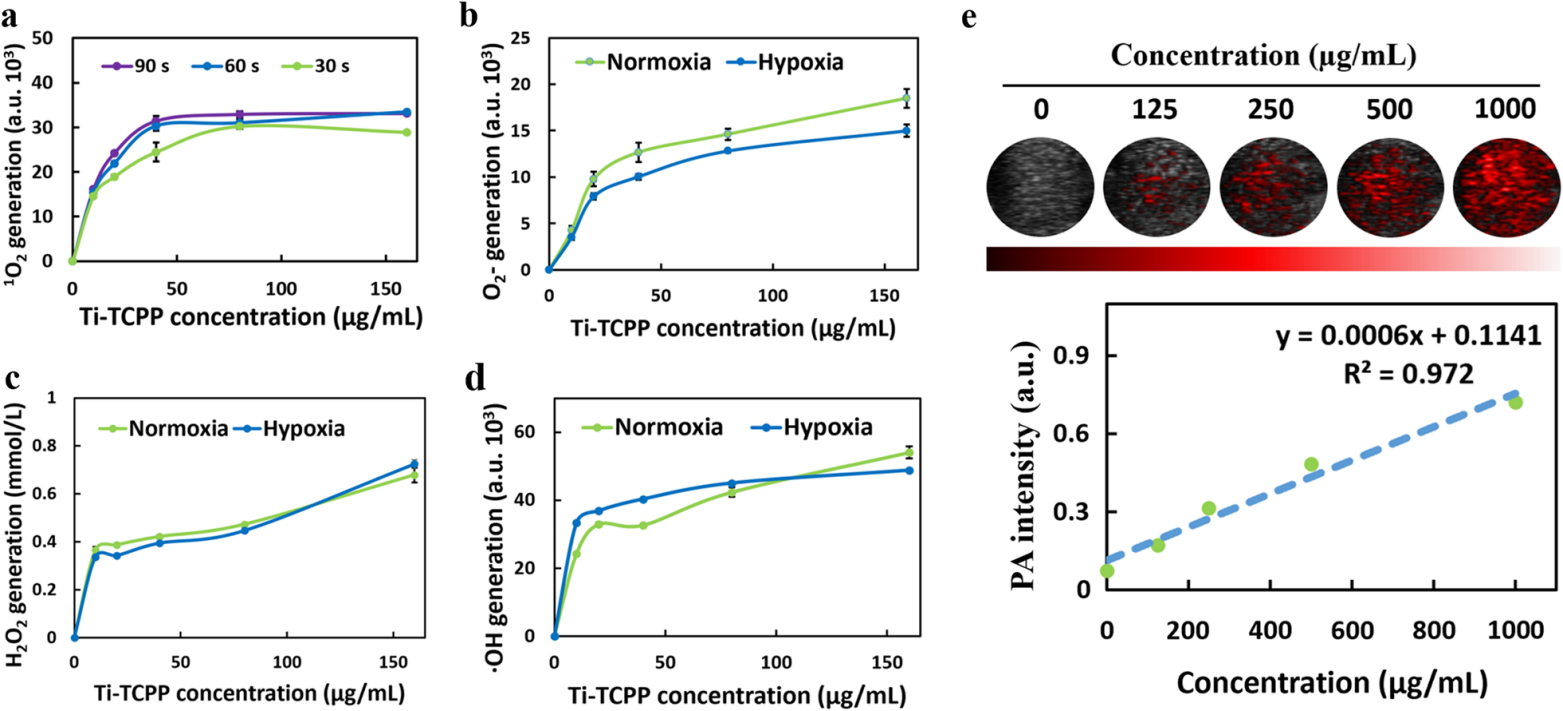
Concentration-dependent ^1^O_2_ generation (**a**), O_2_ˉ generation (**b**), H_2_O_2_ generation (**c**) and ·OH generation (**d**) after US irradiation under normoxic or hypoxic conditions. **e** Normalized intensity of photoacoustic signal versus the concentration of Ti-TCPP MOF solution

Next, we explored the ability of Ti-TCPP MOF preparations to generate ROS. A singlet oxygen sensor green (SOSG) probe was utilized to assess ^1^O_2_ generation following US irradiation [45], revealing an increase in ^1^O_2_ signal in a time- and dose-dependent manner (Fig. 2a), and in a power density-dependent fashion (Additional file 1: Fig. S7a). Rapid increases in SOSG absorbance in Ti-TCPP MOF-containing solutions were consistent with robust and efficient ^1^O_2_ generation. ROS levels produced in an oxygen-independent manner were also assessed, including O_2_^−^ as determined with a dihydrorhodamine 123 (DHR 123) assay kit (Fig. 2b), H_2_O_2_ as measured with a hydrogen peroxide assay kit (Fig. 2c and Additional file 1: S7b), and ·OH as measured via APF assay (Fig. 2d). Upon US irradiation, characteristic absorption values consistent with O_2_^−^, H_2_O_2,_ and ·OH generation gradually increased with Ti-TCPP MOF concentration, consistent with the utility of Ti-TCPP MOF as an effective sonosensitizer capable of simultaneously generating O_2_^−^, H_2_O_2_, and ·OH. The generation of these three ROS species via type I SDT was further verified by conducting these experiments in a hypoxic setting, revealing no apparent changes in O_2_^−^, H_2_O_2_, or ·OH generation. Together, these findings suggested that Ti-TCPP MOF can be utilized as a promising sonosensitizer in hypoxic solid tumors such as pancreatic carcinoma.

Photoacoustic (PA) signal was first assessed in vitro under 680–900 nm pulse laser irradiation, revealing a robust PA signal (Additional file 1: Fig. S8). Then the PA signal at different concentrations of Ti-TCPP MOF under 710 nm pulse laser was calculated to confirm linearity (Fig. 2e), further supporting the promising PA properties of this MOF platform and suggesting that it can be utilized for PA imaging.

### Assessment of Ti-TCPP subcellular localization and antitumor activity

**Fig. 3:**
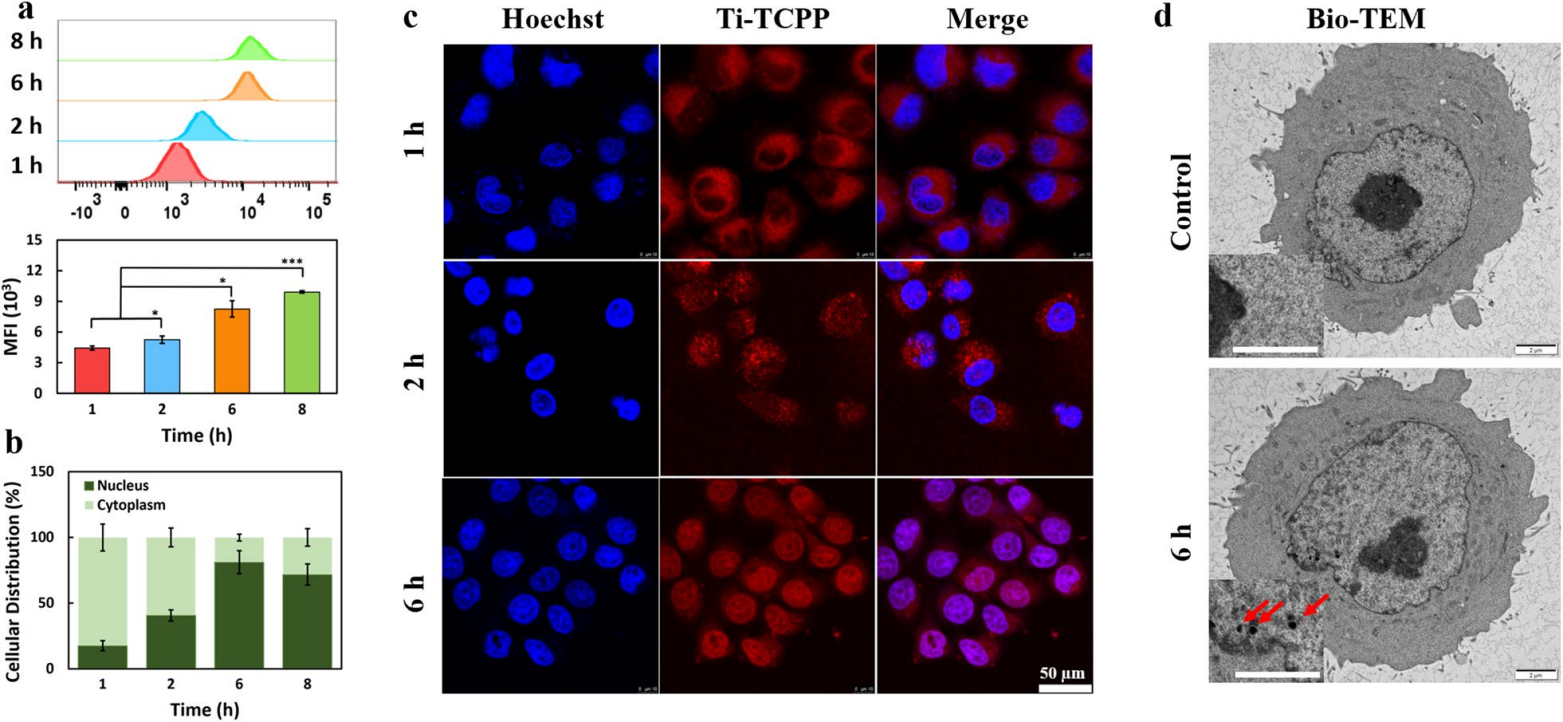
Cellular uptake and nuclear localization of Ti-TCPP MOF in BxPC-3 cells. Flow cytometry (**a**), ICP analysis (**b**) and confocal images (**c**) of BxPC-3 cells after incubation with Ti-TCPP MOF at different time periods. Scale bar: 20 μm. (n = 3). (**d**) Bio-TEM images of BxPC-3 cells before and 6 h after incubation with Ti-TCPP MOF. Scale bar: 2 µm. Red arrows denote Ti-TCPP MOF. *P < 0.05, ***P < 0.001

Next, flow cytometry was used to explore Ti-TCPP MOF uptake by tumor cells, measuring mean fluorescence intensity (MFI) values over time. This analysis revealed a time-dependent increase in Ti-TCPP MOF uptake (Fig. 3a), with rapid increases in MFI values over the first six hours followed by slower increases over the following 2 h. ICP-OES and LSCM were next used to assess the localization of Ti-TCPP MOF within these tumor cells. At 6 h post-treatment, ICP-OES analyses revealed that 81.2% of detected Ti was present in the nucleus of cells with the remaining content being present in the cytoplasm (Fig. 3b). LCSM was then conducted to confirm the nucleus-targeted activity of this MOF platform (Fig. 3c), with Hoechst being used to label BxPC-3 cell nuclei. A small quantity of Ti-TCPP MOFs was detectable in the nuclei of cells within a 2 h treatment period, and such nuclear accumulation rose over time before peaking at 6 h post-treatment, consistent with the ICP-OES results. Bio-TEM examination also revealed the accumulation of Ti-TCPP MOF in the BxPC-3 cells after a 6 h incubation (Fig. 3d), further indicating the successful loading of Ti-TCPP MOFs. Together, these results demonstrated that this ultra-small Ti-TCPP MOF was readily internalized into the nuclei of pancreatic tumor cells. Such passive nuclear targeting may be attributable to the small particle size of this MOF and to changes in zeta potential. Exogenous nanoparticles < 9 nm in size have previously been reported to freely enter the nucleus through nuclear pore complexes (NPCs) [46, 47], and decreasing pH values can lead to a change in zeta potential values from negative to positive [37, 48].

**Fig. 4:**
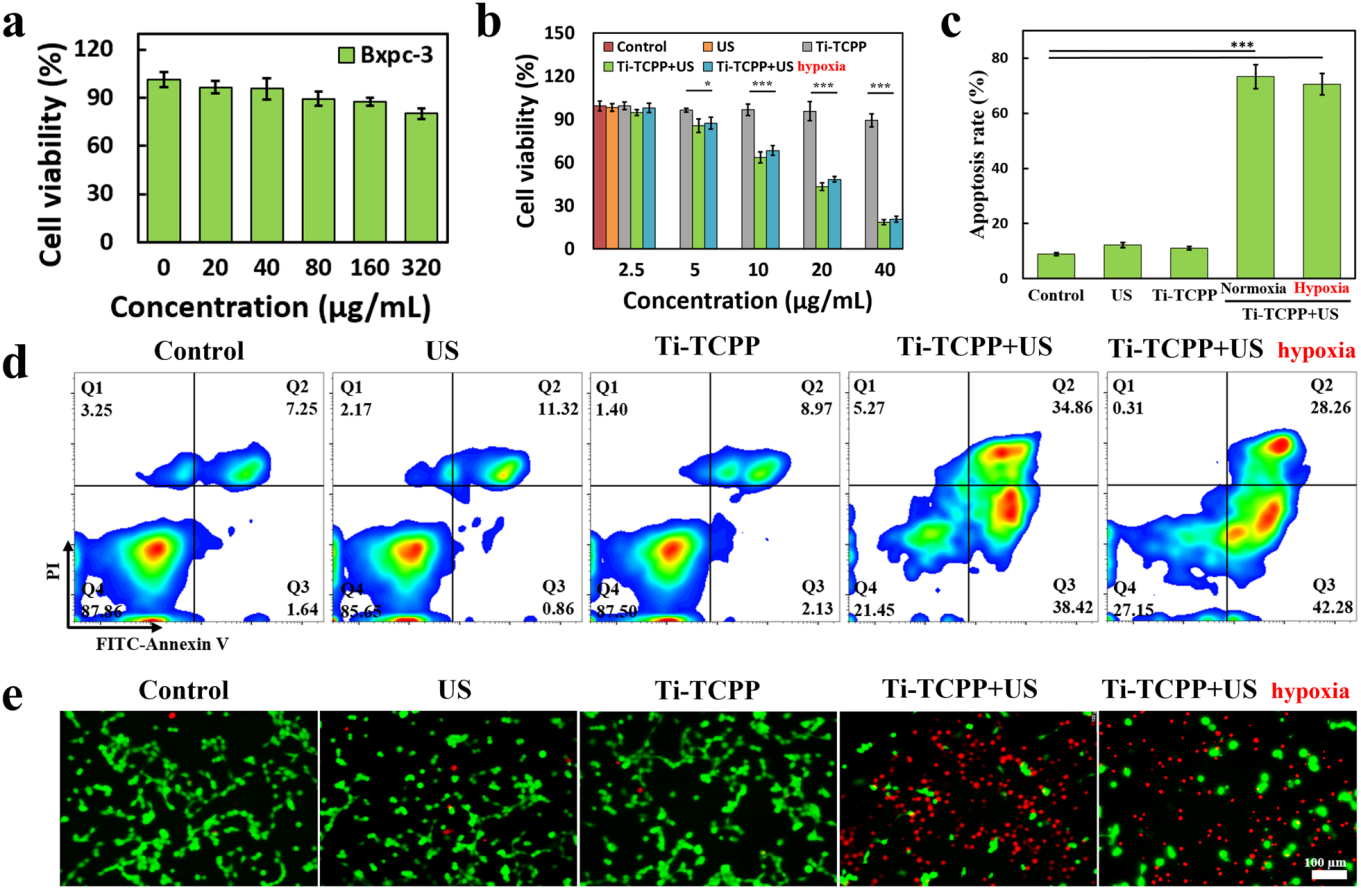
**a** BxPC-3 cell viability after incubation with different concentrations of Ti-TCPP MOF for 24 h (n = 3). **b** Viability of BxPC-3 cells incubated with Ti-TCPP MOF for 6 h, then subjected to US irradiation (0.5 W cm^−2^, 1 MHz, 50% duty cycle, 1 min) and incubated for an additonal 18 h (n = 3). **c**, **d** Flow cytometry analysis of cells after various treatments (n = 3). **e** Live (green) and dead (red) cell staining after various treatments. Scale bar: 100 μm. *P < 0.05, ***P < 0.001

A CCK-8 kit was next utilized to assess Ti-TCPP MOF biocompatibility, revealing no apparent toxicity when either tumor or normal cells (BxPC-3, Panc02, and hTERT-HPNE cell lines) after treatment for 24 h with a range of concentrations (320, 160, 80, 40, or 20 µg mL^−1^) (Fig. 4a and Additional file 1: Fig. S9). Similarly, US irradiation alone had no adverse effect on BxPC-3 cells (Additional file 1: Fig. S10). When BxPC-3 cells were treated with Ti-TCPP MOF + US irradiation, we observed substantial ROS generation in tumor cells (Additional file 1: Fig. S11) and cell proliferation was inhibited in a dose-dependent manner at a US power of 0.5 W cm^−2^ (1 MHz, 50% duty cycle, 1 min) (Fig. 4b). At Ti-TCPP MOF concentrations above 20 µg mL^−1^, cell survival rates fell below 50% under both normoxic and hypoxic conditions, indicating that Ti-TCPP MOF-induced SDT exhibits good therapeutic efficacy when used to kill pancreatic cancer cells in vitro*.* A subsequent flow cytometry analysis similarly confirmed the antitumor activity of this treatment approach, with 73.28% and 70.54% of BxPC-3 cells exhibiting apoptotic cell death following Ti-TCPP + US treatment under normoxic and hypoxic conditions, respectively (Fig. 4c, d), consistent with the results of the CCK-8 assay. A Calcein-AM/PI dual-staining kit was also used to confirm cell viability, revealing no significant cell death in the control, Ti-TCPP, or US treatment groups, whereas the majority of cells in the Ti-TCPP + US group were dead (Fig. 4e). Together, these results confirmed the robust cytotoxicity of our nucleus-targeted SDT treatment strategy both in normoxic and hypoxic environments.

### Analysis of the mechanistic basis for nucleus-targeted SDT

**Fig. 5:**
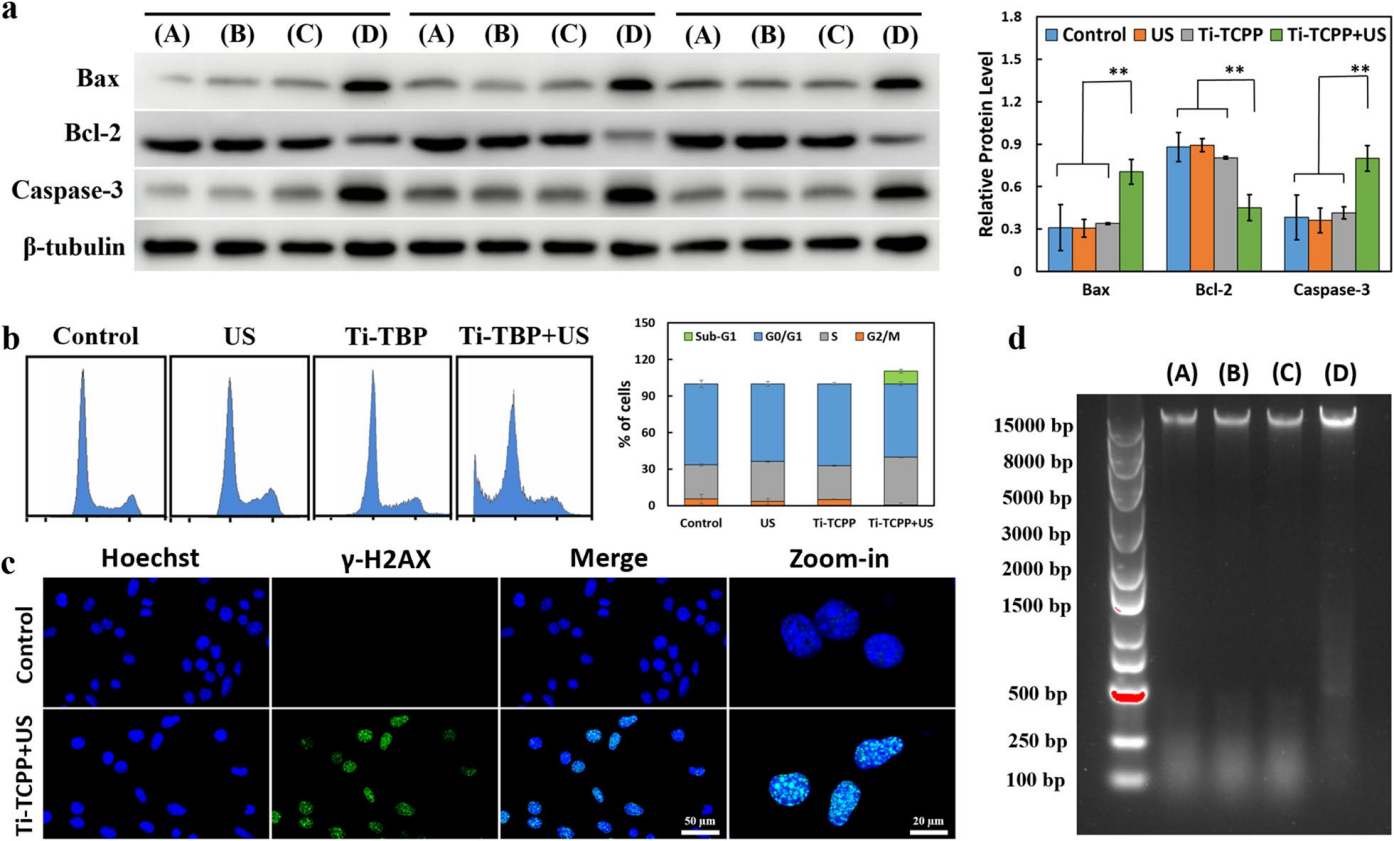
**a** Western blotting analysis of Bax, Bcl-2, and Caspase 3 in BxPC-3 cells incubated under various treatment conditions. β-tubulin was used as an internal control. ImageJ was used to quantify protein levels. Data are means ± S.D. (n = 3). **P < 0.01. **b** Cell cycle progression was evaluated by staining cancer cells with PI and was assessed via flow cytometry after various treatments (n = 3). **c** Confocal images of cancer cells in which the nuclei were stained blue with Hoechst and the γ-H2AX foci bright green following nuclear-targeting Ti-TCPP MOF treatment and US irradiation. **d** A DNA ladder assay was used to evaluate DNA damage after nucleus-targeted SDT therapy. **A** Control, **B** US, **C** Ti-TCPP, **D** Ti-TCPP + US

To understand the mechanisms underlying the efficacy of our nucleus-targeted SDT strategy, we next conducted western blotting, confocal microscopy, cell cycle progression and DNA fragmentation assays. Levels of the apoptosis-related Bax, Bcl-2, and Caspase 3 proteins were measured via Western blotting, with β-tubulin as a loading control (Fig. 5a). Following Ti-TCPP + US treatment, Caspase 3 and Bax expression increased whereas Bcl-2 levels declined, resulting in an overall increase in the Bax/Bcl-2 ratio (Additional file 1: Fig. S12). This result was consistent with the induction of apoptotic cell death in response to treatment-induced ROS generation owing to the irreversible damage of DNA and other biomolecules in these highly proliferative cells. Apoptosis can also occur due to a disruption of the cytokinesis process [49]. Cellular proliferation depends upon cell cycle progression, with cells passing through the G0/G1, S, and G2/M phases in sequence [50–52]. Cells that had undergone Ti-TCPP + US treatment exhibited a significant reduction in the frequency of cells in the G2/M phase (p < 0.05), and a slight increase in the number of cells in the S phase (39.29%) relative to control samples (28.08%) (p < 0.05) (Fig. 5b). Apoptotic cell death was increasingly evident at later time points (24 and 48 h), with respective 10.23% and 29.53% increases in the frequency of sub-G1 cell populations (Additional file 1: Fig. S13). These results indicated that nucleus-targeted SDT treatment can induce both apoptosis and cell cycle arrest at the S phase in tumor cells, thereby inducing mitotic catastrophe.

DNA fragmentation is a hallmark of apoptosis [53–55] and we thus utilized DNA ladder assays and confocal imaging to assess BxPC-3 cells for DNA double-strand breaks (DSBs) [56–58]. As shown in Fig. 5c and Additional file 1: Fig. S14, bright green DSB-associated γ-H2AX foci were evident in the nuclei of cells in the Ti-TCPP + US treatment group, with Hoechst used as a nuclear counterstain. Such DNA damage was also confirmed in a DNA ladder assay (Fig. 5d), wherein Ti-TCPP + US treatment induced DNA cleavage and the formation of a DNA fragment ladder that was absent in samples treated via US or Ti-TCPP alone. We, therefore, concluded that this combination treatment approach can induce DSB formation, likely explaining the observed cell cycle arrest and subsequent apoptotic death observed above. Both cell cycle arrest and apoptosis are effective approaches to eliminating cancer cells, highlighting the value of our nucleus-targeted SDT antitumor therapeutic strategy.

### In vivo biocompatibility of Ti-TCPP MOF

**Fig. 6:**
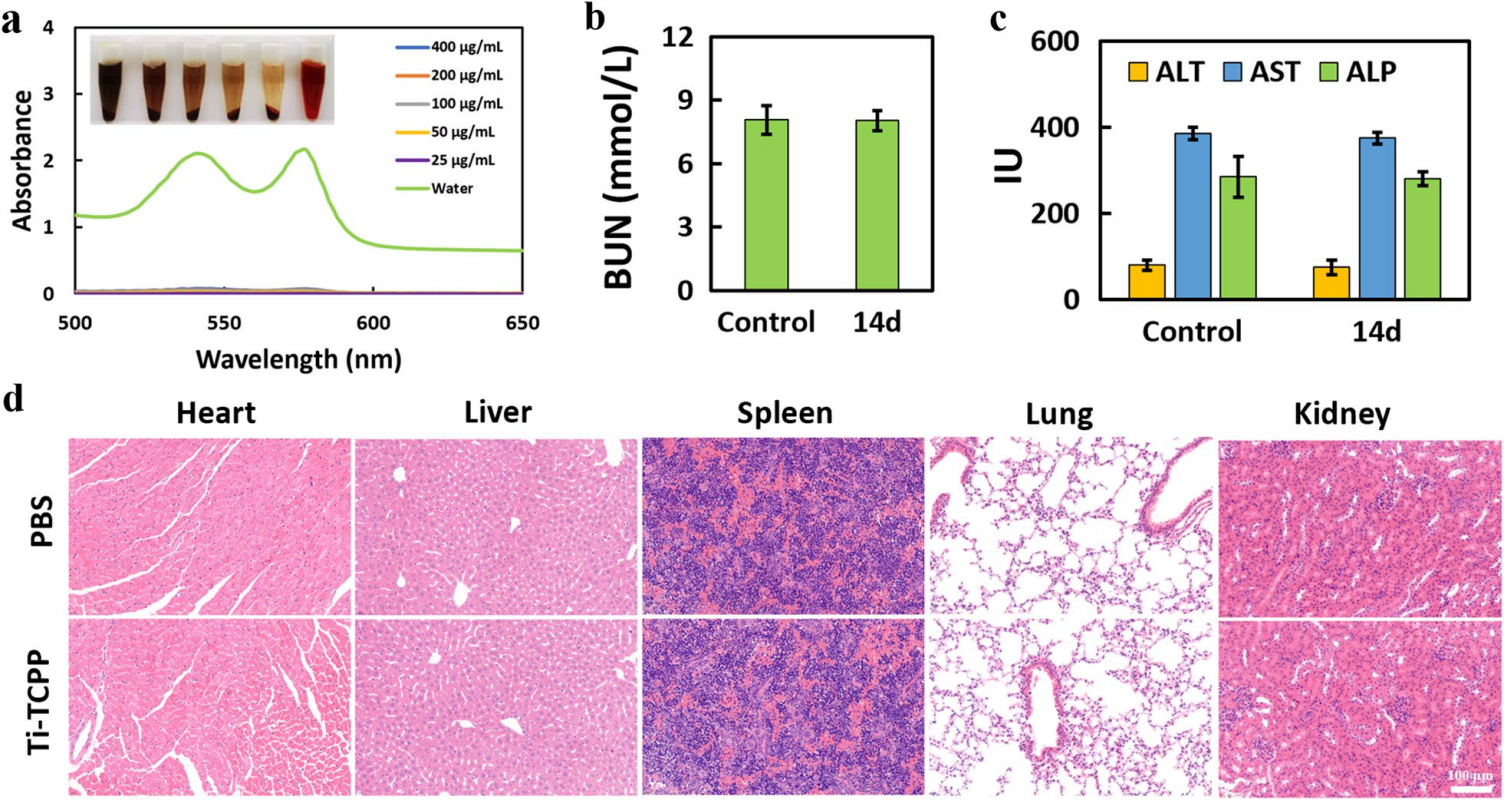
Evaluation of Ti-TCPP MOF in vivo biosafety. **a** Different concentrations of Ti-TCPP MOF were used in a hemolysis assay. The inset images are of samples following centrifugation after incubation of RBCs with Ti-TCPP MOF (400, 200, 100, 50, or 25 µg mL^−1^) or water, respectively (n = 3). **b** Blood urea nitrogen (BUN) levels in healthy mice 14 days post-Ti-TCPP MOF injection (i.v.) (n = 3). **c** Serum aspartate aminotransferase (AST), alanine aminotransferase (ALT), albumin, and alkaline phosphatase (ALP) levels in healthy mice at 14 days post-Ti-TCPP MOF injection (i.v.) (n = 3). **d** H&E-stained images of major organs from healthy control mice 14 days after the i.v. injection of PBS and Ti-TCPP MOF (Scale Bar = 100 µm). Ti-TCPP MOF was injected at a dose of 20 mg/kg

We next evaluated the biosafety of our Ti-TCPP MOF platform by conducting an in vitro hemolysis assay wherein different Ti-TCPP MOF concentrations were combined with murine primary red blood cells. Relative to control samples treated with water, no apparent RBC lysis was observed in the other treatment groups, consistent with good biocompatibility (Fig. 6a and Additional file 1: Fig. S15). We then evaluated the in vivo toxicity of Ti-TCPP MOF preparations by injecting them into healthy nude mice, with PBS serving as a control. No significant weight loss (Additional file 1: Fig. S16) or behavioral changes were observed over 14 days following injection, nor did routine blood (Additional file 1: Fig. S17), kidney (Fig. 6b), liver function analyses (Fig. 6c) or H&E staining of primary organs reveal any treatment-related changes relative to control animals (Fig. 6d). Together these data indicated that Ti-TCPP MOF exhibits a high degree of biosafety and will not cause significant treatment-related toxicity.

### Assessment of Ti-TCPP MOF in vivo distribution and pharmacokinetics

**Fig. 7:**
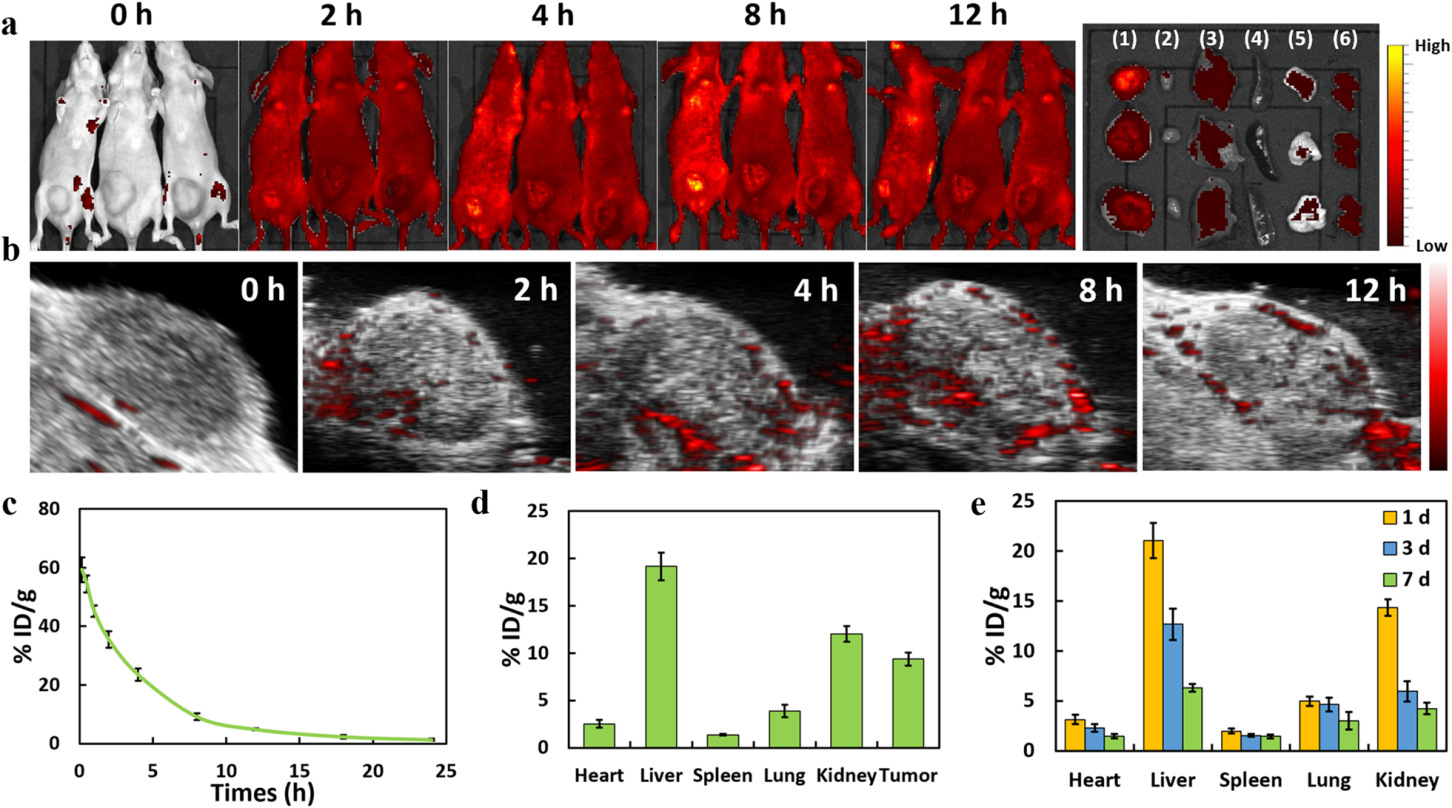
**a** In vivo fluorescence images at different time points post-injection of Ti-TCPP MOF, and of various organs and tumors at 12 h post-injection. **1** tumor, **2** heart, **3** liver, **4** spleen, **5** lung and **6** kidney. **b** In vivo photoacoustic images at different time points post-Ti-TCPP MOF injection. Grayscale images represent ultrasound images, and colored images represent photoacoustic images. **c** Circulating Ti-TCPP MOF levels after i.v. injection, as assessed via ICP-OES. Ti-TCPP MOF pharmacokinetics followed a two-compartment model (n = 3). **d** Ti-TCPP MOF biodistribution in BxPC-3 tumor-bearing mice at 8 h post-i.v. injection (n = 3). **e** Time-dependent distribution of Ti in the primary organs of healthy mice after the i.v. injection of Ti-TCPP MOF (n = 3)

To more reliably assess the intratumoral accumulation of Ti-TCPP MOF in vivo*,* fluorescent and PA imaging were conducted at a range of time points (0, 2, 4, 8, and 12 h) following intravenous injection into mice bearing BxPC-3 tumors. Fluorescence increased in a time-dependent manner, reaching maximal fluorescence at 8 h post-injection and with a strong signal remaining evident at the 12 h time point (Fig. 7a and Additional file 1: Fig. S18a). Analyses of primary organs from these mice at 12 h post-injection further supported the accumulation of Ti-TCPP MOF within tumors (Additional file 1: Fig. S18b). Analyses of the in vivo PA signal yielded comparable results to those of fluorescence intensity analyses (Fig. 7b).

Next, ICP-OES was used to measure Ti concentrations in the blood and major organs of these mice, revealing a gradual reduction in Ti-TCPP MOF concentrations in the blood within 24 h with a half-life of 5.09 ± 0.38 h calculated using a two-compartment model (Fig. 7c). This relatively long half-life facilitated the passive accumulation of Ti-TCPP MOF within tumors owing to the enhanced permeability and retention effect, with an accumulation of 9.37 ± 0.68% ID/g at 8 h post-injection within murine tumors (Fig. 7d). Ti levels within major organs were assessed over a 7-day period following treatment, revealing relatively low concentrations within 3 days, and with nearly complete clearance after one week (Fig. 7e). These results suggest that while ultra-small Ti-TCPP MOF can efficiently accumulate within tumors, it can be readily metabolized by the liver and kidney, thus reducing its overall accumulation in the body, and facilitating excellent biocompatibility and safety.

### In vivo antitumor efficacy

**Fig. 8:**
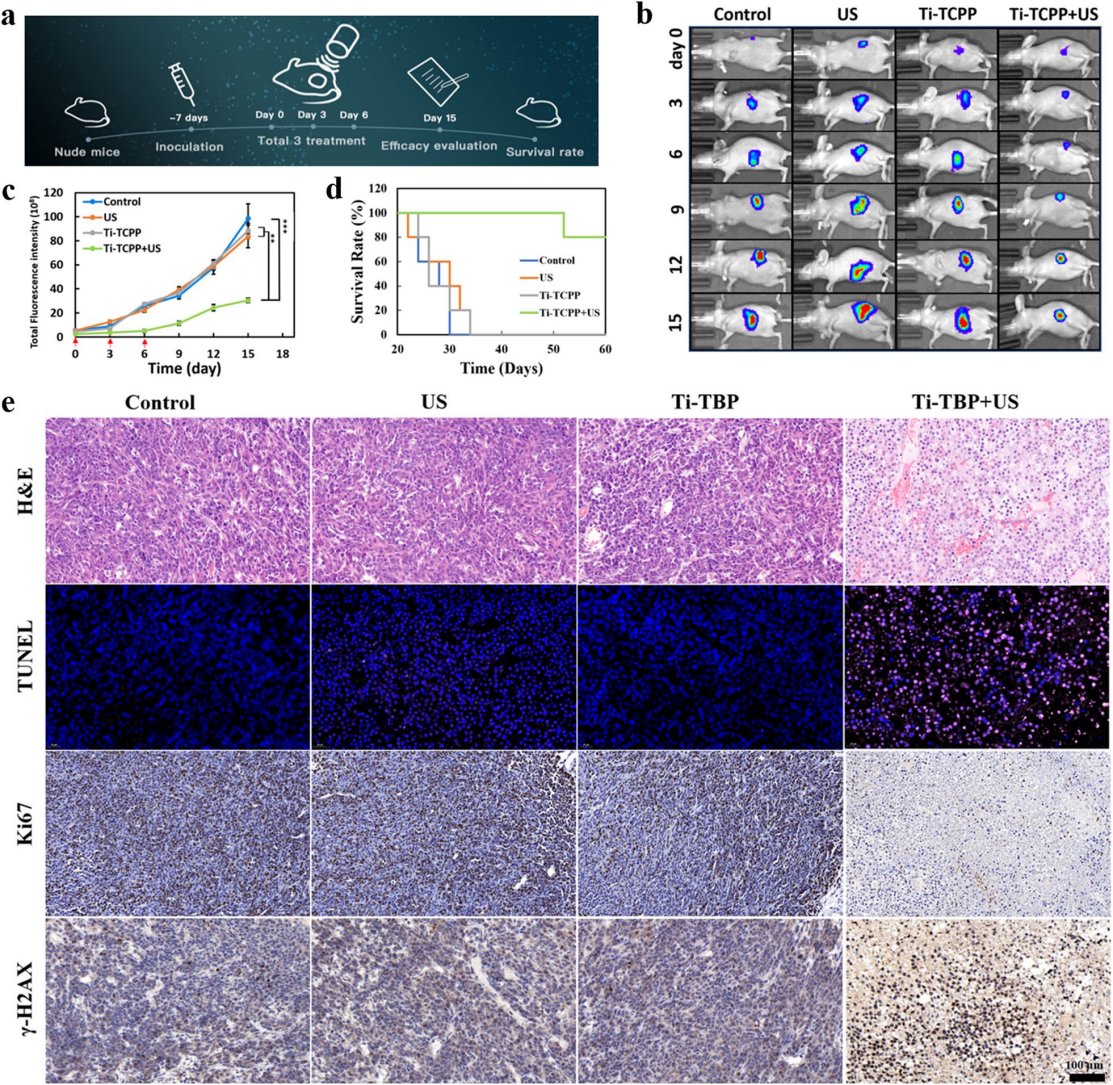
In vivo antitumor evaluation. **a** Schematic illustration of the therapy process. **b** Fluorescence images of tumor-bearing mice after various treatments. **c** Tumor fluorescence intensity changes and **d** survival rates of tumor-bearing mice after different treatments. Data are means ± SD (n = 5). **e** H&E, TUNEL, Ki67, and γ-H2AX staining of tumors 15 day after various treatments (Scale bar = 100 µm)

After establishing an orthotopic model of murine pancreatic cancer, mice were randomly assigned to four treatment groups, with three total treatments, and tumor weight, body weight and fluorescence images being captured every three days (Fig. 8a). A 2.0 cm-thick US gel pad was utilized to reduce thermal effects during US irradiation.

Mice treated with Ti-TCPP MOF + US exhibited significant inhibition of tumor growth, whereas tumor-associated fluorescent signal rapidly increased in intensity over time in the other three treatment groups (Fig. 8b, c). Mice in the Ti-TCPP + US group survived for over 60 days, whereas mice in the other three groups survived a maximum of 34 days (Fig. 8d). These results were consistent with the efficacy of our nucleus-targeted SDT approach. Importantly, no significant decreases in murine body weight were observed over the 15-day treatment period (Additional file 1: Fig. S19), nor were any significant changes observed upon histopathological examination of H&E-stained heart, liver, spleen, lung, and kidney tissue samples from the mice in any treatment group, consistent with the safety of this therapeutic strategy (Additional file 1: Fig. S20). Tumors were then collected and subjected to H&E, TUNEL, Ki67, and γ-H2AX staining to evaluate the efficacy of this SDT treatment approach (Fig. 8e). H&E-stained tumor sections revealed clear nuclear fragmentation and a reduction in nucleus size in cells in the Ti-TCPP MOF + US group. The TUNEL assay has been widely used to detect apoptotic cells in tumor tissues while Ki67 is a sensitive biomarker for cell proliferation [59, 60]. Consistent with H&E staining results, TUNEL staining confirmed that this treatment combination greatly increased the rate of tumor cell apoptosis, while Ki67 staining indicated a reduction in tumor cell proliferation following Ti-TCPP MOF + US treatment relative to other treatments. Intranuclear γ-H2AX levels were also increased in tumor cells from mice treated with Ti-TCPP MOF + US, consistent with severe DNA damage. Overall, these results thus confirmed the direct antitumor activity of Ti-TCPP MOF as a nucleus-targeted sonosensitizing agent.

## Conclusions

In conclusion, we developed an ultra-small Ti-TCPP MOF for effective nucleus-targeted SDT. These preparations were able to intrinsically target cellular nuclei without additional modification, therein destroying DNA, inducing S phase cell cycle arrest, and thereby driving apoptotic cell death. This MOF platform was able to efficiently generate ROS, thereby facilitating both type I and type II SDT in an oxygen-independent manner. This activity was consistent with a promising approach to the treatment of hypoxic solid tumors. In in vivo experiments, Ti-TCPP MOF was almost fully cleared within 7 days of treatment without any evidence of hematological or histopathological toxicity. As such, our nucleus-targeted Ti-TCPP MOF platform represents a novel and efficient approach to facilitating SDT in response to low-intensity ultrasound.

## Supplementary Information

**Additional file 1: Fig. S1.** Structural formula and synthesis method of ultra-small Ti-TCPP MOF. **Fig. S2.** TEM images and DLS measurement of Ti-TCPP MOF in PBS for 21 days (a) and FBS for 7 days (b) at 4 ℃. Scale bar: 50 nm. **Fig. S3.** XRD pattern of Ti-TCPP MOF in PBS for 21 days at 4 ℃. **Fig. S4.** TEM images and DLS measurement of Ti-TCPP MOF in PBS (a) and FBS for 3 days (b) at 37 ℃. Scale bar: 50 nm. **Fig. S5.** TEM images and DLS measurement of Ti-TCPP MOF in PBS after US irradiation (0.5 W cm^−2^, 1 MHz, 50% duty cycle, 1 min). Scale bar: 50 nm. **Fig. S6.** N_2_ adsorption/desorption result of Ti-TCPP MOF. **Fig. S7.** (a) Power-dependent ^1^O_2_ generation after US irradiation under normoxia conditions. (b) A standard curve of H_2_O_2_ generation measured by multiscan spectrum. **Fig. S8.** The PA signal of Ti-TCPP MOF under 680–900 nm pulse laser irradiation in vitro. **Fig. S9.** Cell viability of Panc02 (a) and hTERT-HPNE (b) cells after incubated with Ti-TCPP MOF for 24 h. **Fig. S10.** Cell viability of BxPC-3 cells after US irradiation (1 MHz, 50% duty cycle, 1 min) for 24 h. **Fig. S11.** Detection of ROS generation by DCFH-DA kit in tumor cells. **Fig. S12.** The quantified levels of Bax/Bcl-2 was analyzed by Image J software. The data were expressed as mean ± S.D. (n = 3). ***P < 0.001. **Fig. S13.** Cell cycle analysis determined by flow cytometry (a) and statistical analysis of sub-G1 phase (b) after treatment with Ti-TCPP + US at different time period (n = 3). ***p < 0.001. **Fig. S14.** Confocal images of cancer cells in which the nuclei were stained blue with Hoechst and the γ-H2AX foci bright green following nuclear-targeting Ti-TCPP MOF treatment or US irradiation. **Fig. S15.** Hemolysis coefficient after incubation of RBCs with Ti-TCPP MOF (400, 200, 100, 50, or 25 µg mL^−1^) or water, respectively (n = 3). The hemolysis coefficient of Ti-TCPP MOF was less than 5%, indicating the good biocompatibility of Ti-TCPP MOF. **Fig. S16.** The body weight changes of healthy mice treated by PBS or Ti-TCPP MOF during 14 days. **Fig. S17.** Blood routine analysis. Blood levels of WBC, RBC, HGB, HCT, MCHC, PLT, MCV and MCH of health mice after 14 d post injection (i.v.) of Ti-TCPP MOF, PBS was set as control. **Fig. S18.** (a) Statistics analysis of tumor fluorescence intensity after i.v. Ti-TCPP MOF injection at different time period (n = 3). (b) Statistics analysis of fluorescence intensity of main organs 12 h after i.v. Ti-TCPP MOF injection (n = 3). **Fig. S19.** The body weight of mice after various treatment for 15 days, indicating no acute toxicity to mice major organs. n = 5. **Fig. S20.** Histological analysis of the main organs (heart, liver, spleen, lung and kidney) of un-treated mice (control) and mice treated with US, Ti-TCPP MOF and Ti-TCPP MOF + US for 15 days. Scale bar = 100 µm.

## Abbreviations

MOF: Metal–organic framework
ROS: Reactive oxygen species
NIR: Near-infrared
DMF: N,N-dimethylformamide
H_4_TBP: 5,10,15,20-Tetra(p-benzoato)porphyrin
AcOH: Acetic acid
DMSO: Dimethyl sulfoxide
^1^O_2_: Singlet oxygen
SOSG: Singlet oxygen sensor green
O2ˉ: Superoxide
H_2_O_2_: Hydrogen peroxide
·OH: Hydroxyl radical
APF: Aminophenyl fluorescein
CLSM: Confocal laser scanning microscopy
ICP-OES: Inductively coupled plasma optical emission spectrometry
CCK-8: Cell counting kit-8
DHR 123: Dihydrorhodamine 123
PA: Photoacoustic
MFI: Mean fluorescence intensity
NPCs: Nuclear pore complexes
DSBs: DNA double-strand breaks

## References

[1] Pan X, Wang W, Huang Z (2020). MOF-derived double-layer hollow nanoparticles with oxygen generation for multimodal imaging-guided sonodynamic therapy. Angew Chem Int Ed Engl.

[2] Zhang Y, Bi L, Hu Z (2020). Hematoporphyrin monomethyl ether-mediated sonodynamic therapy induces A-253 cell apoptosis. Oncol Lett.

[3] Chang N, Qin D, Wu P (2019). IR780 loaded perfluorohexane nanodroplets for efficient sonodynamic effect induced by short-pulsed focused ultrasound. Ultrason Sonochem.

[4] Ma A, Chen H, Cui Y (2019). Metalloporphyrin complex-based nanosonosensitizers for deep-tissue tumor theranostics by noninvasive sonodynamic therapy. Small.

[5] Lafond M, Yoshizawa S, Umemura SI (2019). Sonodynamic therapy: advances and challenges in clinical translation. J Ultrasound Med.

[6] Prescott M, Mitchell J, Totti S (2018). Sonodynamic therapy combined with novel anti-cancer agents, sanguinarine and ginger root extract: synergistic increase in toxicity in the presence of PANC-1 cells in vitro. Ultrason Sonochem.

[7] Lu K, He C, Guo N (2016). Chlorin-based nanoscale metal-organic framework systemically rejects colorectal cancers via synergistic photodynamic therapy and checkpoint blockade immunotherapy. J Am Chem Soc.

[8] Park J, Jiang Q, Feng D (2016). Size-controlled synthesis of porphyrinic metal-organic framework and functionalization for targeted photodynamic therapy. J Am Chem Soc.

[9] Brown JM, Wilson WR (2004). Exploiting tumour hypoxia in cancer treatment. Nat Rev Cancer.

[10] Zhao H, Zhao B, Li L (2020). Biomimetic decoy inhibits tumor growth and lung metastasis by reversing the drawbacks of sonodynamic therapy. Adv Healthc Mater.

[11] Zhu P, Chen YShi J, (2018). Nanoenzyme-augmented cancer sonodynamic therapy by catalytic tumor oxygenation. ACS Nano.

[12] Koong AC, Mehta VK, Le QT (2000). Pancreatic tumors show high levels of hypoxia. Int J Radiat Oncol Biol Phys.

[13] Li E, Sun Y, Lv G (2019). Sinoporphyrin sodium based sonodynamic therapy induces anti-tumor effects in hepatocellular carcinoma and activates p53/caspase 3 axis. Int J Biochem Cell Biol.

[14] Vaupel P, Hockel M, Mayer A (2007). Detection and characterization of tumor hypoxia using pO2 histography. Antioxid Redox Signal.

[15] Li X, Peng XH, Zheng BD (2018). New application of phthalocyanine molecules: from photodynamic therapy to photothermal therapy by means of structural regulation rather than formation of aggregates. Chem Sci.

[16] Juarranz A, Jaen P, Sanz-Rodriguez F (2008). Photodynamic therapy of cancer. Basic principles and applications. Clin Transl Oncol.

[17] Rengeng L, Qianyu Z, Yuehong L (2017). Sonodynamic therapy, a treatment developing from photodynamic therapy. Photodiagnosis Photodyn Ther.

[18] Nesbitt H, Sheng Y, Kamila S (2018). Gemcitabine loaded microbubbles for targeted chemo-sonodynamic therapy of pancreatic cancer. J Control Release.

[19] Chen Y, Du M, Yu J (2020). Nanobiohybrids: a synergistic integration of bacteria and nanomaterials in cancer therapy. BIO Integration.

[20] Pan L, Liu JShi J, (2018). Cancer cell nucleus-targeting nanocomposites for advanced tumor therapeutics. Chem Soc Rev.

[21] Cheng H, Fan JH, Zhao LP (2019). Chimeric peptide engineered exosomes for dual-stage light guided plasma membrane and nucleus targeted photodynamic therapy. Biomaterials.

[22] Liu Z, Qiu K, Liao X (2020). Nucleus-targeting ultrasmall ruthenium(iv) oxide nanoparticles for photoacoustic imaging and low-temperature photothermal therapy in the NIR-II window. Chem Commun.

[23] Zhang P, Huang H, Banerjee S (2019). Nucleus-targeted organoiridium-albumin conjugate for photodynamic cancer therapy. Angew Chem Int Ed.

[24] Ma X, Gong N, Zhong L (2016). Future of nanotherapeutics: targeting the cellular sub-organelles. Biomaterials.

[25] Gao X, Zhang J, Huang Z (2017). Reducing interstitial fluid pressure and inhibiting pulmonary metastasis of breast cancer by gelatin modified cationic lipid nanoparticles. ACS Appl Mater Interfaces.

[26] Pan L, He Q, Liu J (2012). Nuclear-targeted drug delivery of TAT peptide-conjugated monodisperse mesoporous silica nanoparticles. J Am Chem Soc.

[27] Li Y, Mei T, Han S (2020). Cathepsin B-responsive nanodrug delivery systems for precise diagnosis and targeted therapy of malignant tumors. Chin Chem Lett.

[28] Cao Y, Wu T, Zhang K (2019). Engineered exosome-mediated near-infrared-II region V2C quantum dot delivery for nucleus-target low-temperature photothermal therapy. ACS Nano.

[29] Zhang C, Jin R, Zhao P (2015). A family of cationic polyamides for in vitro and in vivo gene transfection. Acta Biomater.

[30] Vaidyanathan S, Chen J, Orr BG (2016). Cationic polymer intercalation into the lipid membrane enables intact polyplex DNA escape from endosomes for gene delivery. Mol Pharm.

[31] Zhang T, Jiang Z, Chen L (2020). PCN-Fe(III)-PTX nanoparticles for MRI guided high efficiency chemo-photodynamic therapy in pancreatic cancer through alleviating tumor hypoxia. Nano Res.

[32] Pan X, Bai L, Wang H (2018). Metal-organic-framework-derived carbon nanostructure augmented sonodynamic cancer therapy. Adv Mater.

[33] Zhang K, Meng XD, Cao Y (2018). Metal-organic framework nanoshuttle for synergistic photodynamic and low-temperature photothermal therapy. Adv Funct Mater.

[34] Horcajada P, Gref R, Baati T (2012). Metal–organic frameworks in biomedicine. Chem Rev.

[35] Chen WH, Liao WC, Sohn YS (2018). Stimuli-responsive nucleic acid-based polyacrylamide hydrogel-coated metal–organic framework nanoparticles for controlled drug release. Adv Funct Mater.

[36] Chen WH, Luo GF, Zhang XZ (2019). Recent advances in subcellular targeted cancer therapy based on functional materials. Adv Mater.

[37] Jiang Z, Yuan B, Wang Y (2020). Near-infrared heptamethine cyanine dye-based nanoscale coordination polymers with intrinsic nucleus-targeting for low temperature photothermal therapy. Nano Today.

[38] Wang D, Wu H, Lim WQ (2019). A mesoporous nanoenzyme derived from metal-organic frameworks with endogenous oxygen generation to alleviate tumor hypoxia for significantly enhanced photodynamic therapy. Adv Mater.

[39] Zhang A, Pan S, Zhang Y (2019). Carbon-gold hybrid nanoprobes for real-time imaging, photothermal/photodynamic and nanozyme oxidative therapy. Theranostics.

[40] Xu X (2016). Controllable synthesis of ultra-small metal–organic framework nanocrystals composed of copper (ii) carboxylates. Nanoscale.

[41] Wang H, Yu D, Fang J (2019). Renal-clearable porphyrinic metal–organic framework nanodots for enhanced photodynamic therapy. ACS Nano.

[42] Gao G, Jiang YW, Sun W (2019). Molecular targeting-mediated mild-temperature photothermal therapy with a smart albumin-based nanodrug. Small.

[43] Liu L-H, Qiu W-X, Zhang Y-H (2017). A charge reversible self-delivery chimeric peptide with cell membrane-targeting properties for enhanced photodynamic therapy. Adv Funct Mater.

[44] Lan G, Ni K, Veroneau SS (2019). Titanium-based nanoscale metal-organic framework for type I photodynamic therapy. J Am Chem Soc.

[45] Yang Z, Wang J, Ai S (2019). Self-generating oxygen enhanced mitochondrion-targeted photodynamic therapy for tumor treatment with hypoxia scavenging. Theranostics.

[46] Paine PL, Moore LC, Horowitz SB (1975). Nuclear envelope permeability. Nature.

[47] Yuan Y, Liu C, Qian J (2010). Size-mediated cytotoxicity and apoptosis of hydroxyapatite nanoparticles in human hepatoma HepG2 cells. Biomaterials.

[48] Zhang M, Chen X, Li C (2020). Charge-reversal nanocarriers: an emerging paradigm for smart cancer nanomedicine. J Control Release.

[49] Kang B, Mackey MAEl-Sayed MA, (2010). Nuclear targeting of gold nanoparticles in cancer cells induces DNA damage, causing cytokinesis arrest and apoptosis. J Am Chem Soc.

[50] Zhong Z, Zhou F, Wang D (2018). Expression of KLF9 in pancreatic cancer and its effects on the invasion, migration, apoptosis, cell cycle distribution, and proliferation of pancreatic cancer cell lines. Oncol Rep.

[51] Azorsa DO, Gonzales IM, Basu GD (2009). Synthetic lethal RNAi screening identifies sensitizing targets for gemcitabine therapy in pancreatic cancer. J Transl Med.

[52] Xu H, Cheung IY, Wei XX (2011). Checkpoint kinase inhibitor synergizes with DNA-damaging agents in G1 checkpoint-defective neuroblastoma. Int J Cancer.

[53] Majtnerova P, Rousar T (2018). An overview of apoptosis assays detecting DNA fragmentation. Mol Biol Rep.

[54] Zhou J, Wang Q, Geng S (2019). Construction and evaluation of tumor nucleus-targeting nanocomposite for cancer dual-mode imaging—guiding photodynamic therapy/photothermal therapy. Mater Sci Eng C Mater Biol Appl.

[55] Yumita N, Iwase Y, Nishi K (2012). Involvement of reactive oxygen species in sonodynamically induced apoptosis using a novel porphyrin derivative. Theranostics.

[56] Luan S, Yun X, Rao W (2017). Emamectin benzoate induces ROS-mediated DNA damage and apoptosis in *Trichoplusia* Tn5B1-4 cells. Chem Biol Interact.

[57] Liu S, Cao W, Yu L (2013). Zinc(II) complexes containing bis-benzimidazole derivatives as a new class of apoptosis inducers that trigger DNA damage-mediated p53 phosphorylation in cancer cells. Dalton Trans.

[58] Bian C, Zhang C, Luo T (2019). NADP(+) is an endogenous PARP inhibitor in DNA damage response and tumor suppression. Nat Commun.

[59] Yan X, Yang L, Feng G (2018). Lup-20(29)-en-3beta,28-di-yl-nitrooxy acetate affects MCF-7 proliferation through the crosstalk between apoptosis and autophagy in mitochondria. Cell Death Dis.

[60] Wu J, Bremner DH, Niu S (2018). Chemodrug-gated biodegradable hollow mesoporous organosilica nanotheranostics for multimodal imaging-guided low-temperature photothermal therapy/chemotherapy of cancer. ACS Appl Mater Interfaces.

