# Supplementary material for: Intrinsic nucleus-targeted ultra-small metal–organic framework for the type I sonodynamic treatment of orthotopic pancreatic carcinoma
Source: J Nanobiotechnology. 2021 Oct 12;19:315. doi: 10.1186/s12951-021-01060-7 (PMC8507249; doi:10.1186/s12951-021-01060-7)
Supplement: Supplementary file 1 — Additional file 1: Fig. S1. Structural formula and synthesis method of ultra-small Ti-TCPP MOF. Fig. S2. TEM images and DLS measurement of Ti-TCPP MOF in PBS for 21 days (a) and FBS for 7 days (b) at 4 ℃. Scale bar: 50 nm. Fig. S3. XRD pattern of Ti-TCPP MOF in PBS for 21 days at 4 ℃. Fig. S4. TEM images and DLS measurement of Ti-TCPP MOF in PBS (a) and FBS for 3 days (b) at 37 ℃. Scale bar: 50 nm. Fig. S5. TEM images and DLS measurement of Ti-TCPP MOF in PBS after US irradiation (0.5 W cm−2, 1 MHz, 50% duty cycle, 1 min). Scale bar: 50 nm. Fig. S6. N2 adsorption/desorption result of Ti-TCPP MOF. Fig. S7. (a) Power-dependent 1O2 generation after US irradiation under normoxia conditions. (b) A standard curve of H2O2 generation measured by multiscan spectrum. Fig. S8. The PA signal of Ti-TCPP MOF under 680–900 nm pulse laser irradiation in vitro. Fig. S9. Cell viability of Panc02 (a) and hTERT-HPNE (b) cells after incubated with Ti-TCPP MOF for 24 h. Fig. S10. Cell viability of BxPC-3 cells after US irradiation (1 MHz, 50% duty cycle, 1 min) for 24 h. Fig. S11. Detection of ROS generation by DCFH-DA kit in tumor cells. Fig. S12. The quantified levels of Bax/Bcl-2 was analyzed by Image J software. The data were expressed as mean ± S.D. (n = 3). ***P < 0.001. Fig. S13. Cell cycle analysis determined by flow cytometry (a) and statistical analysis of sub-G1 phase (b) after treatment with Ti-TCPP + US at different time period (n = 3). ***p < 0.001. Fig. S14. Confocal images of cancer cells in which the nuclei were stained blue with Hoechst and the γ-H2AX foci bright green following nuclear-targeting Ti-TCPP MOF treatment or US irradiation. Fig. S15. Hemolysis coefficient after incubation of RBCs with Ti-TCPP MOF (400, 200, 100, 50, or 25 µg mL−1) or water, respectively (n = 3). The hemolysis coefficient of Ti-TCPP MOF was less than 5%, indicating the good biocompatibility of Ti-TCPP MOF. Fig. S16. The body weight changes of healthy mice treated by PBS or Ti-TCPP MOF during [file 12951_2021_1060_MOESM1_ESM.docx]

Additional file 1:

**Intrinsic** **Nucleus-Targeted Ultra-Small Metal-Organic Framework for the Type I Sonodynamic Treatment of Orthotopic Pancreatic Carcinoma**

Tao Zhang^1,2^, Yu Sun^1,2^, Jing Cao^1,2^, Jiali Luo^1,2^, Jing Wang^1,2^, Zhenqi Jiang^3,^*, Pintong Huang^1,2,^*

*^1^Department of Ultrasound in Medicine, The Second Affiliated Hospital of Zhejiang University School of Medicine, No.88 Jiefang Road, Shangcheng District, Hangzhou 310009, P.R. China*

*^2^Research Center of Ultrasound in Medicine and Biomedical Engineering, The Second Affiliated Hospital of Zhejiang University School of Medicine, No.88 Jiefang Road, Shangcheng District, Hangzhou 310009, P.R. China.*

*^3^Institute of Engineering Medicine, Beijing Institute of Technology, No. 5, South Street, Zhongguancun, Haidian District, Beijing 100081, P.R. China*

**Experimental Section**

1. Materials and Cell lines: All the starting materials were purchased from Sigma-Aldrich and Fisher (USA), unless otherwise noted, and used without further purification. The human pancreatic carcinoma cells BxPC-3, Murine pancreatic cancer cells Panc02 and immortalized pancreas duct cells hTERT-HPNE were purchased from Shanghai Cell Bank. BxPC-3 cells were routinely cultured in RPMI1640 medium (Gibco) supplemented with 10% FBS (Gibco), Panc02 and hTERT-HPNE in Dulbecco’s modified Eagle’s medium (DMEM) (Gibco) supplemented with 10% and 5% FBS (Gibco), respectively.

2. Apparatus: The morphologies of samples were studied with TEM (JEOL-2100, JEOL, Japan). The DLS and zeta potential of Ti-TCPP MOF were measured at room temperature using a particle analyzer (Nano-ZS, Malvern, England). All fluorescence images were measured using a confocal laser scanning microscopy (CLSM, FV1200, Olympus, Japan). PA imaging was conducted by scanning the excitation wavelength of the PA imaging system (Vevo LAZR, Canada) from 680 to 970 nm. Surface elemental analysis of Ti-TCPP MOF was determined by X-ray photoelectron spectroscopy (XPS, AXIS ULTRA DLD, Shimadzu, Japan). The structural properties of samples were investigated by X-ray diffraction (XRD) using an X-ray powder diffractometer (D8 Discover, Bruker AXS).

3. Flow cytometry cell cycle analysis: BxPC-3 cells were grown in 6-well culture plates overnight. After various treatment same as CCK-8 assay, cells were fixed in 70% ethanol, centrifuged, washed, and resuspended in phosphate buffered saline (PBS). Cells were then treated with 10 μg/mL RNase (Sigma) at 37 °C for 30 min, after which they were stained with 100 μg/mL of propidium iodide (PI), fluorescent DNA stain, for 15 min at room temperature. Then samples were analyzed using the BD LSR II (BD Biosciences) with a 488 nm excitation.

4. Western Blot: After treated consistently with CCK-8 assay, proteins were quantified and loaded onto gels before transfer to PVDF membranes. The primary antibodies including Caspase-3, Bcl-2, Bax and β-tubulin (1/1000, CST) were incubated first, and then incubated with the secondary antibody. At last, ECL reagent was used to detect proteins.


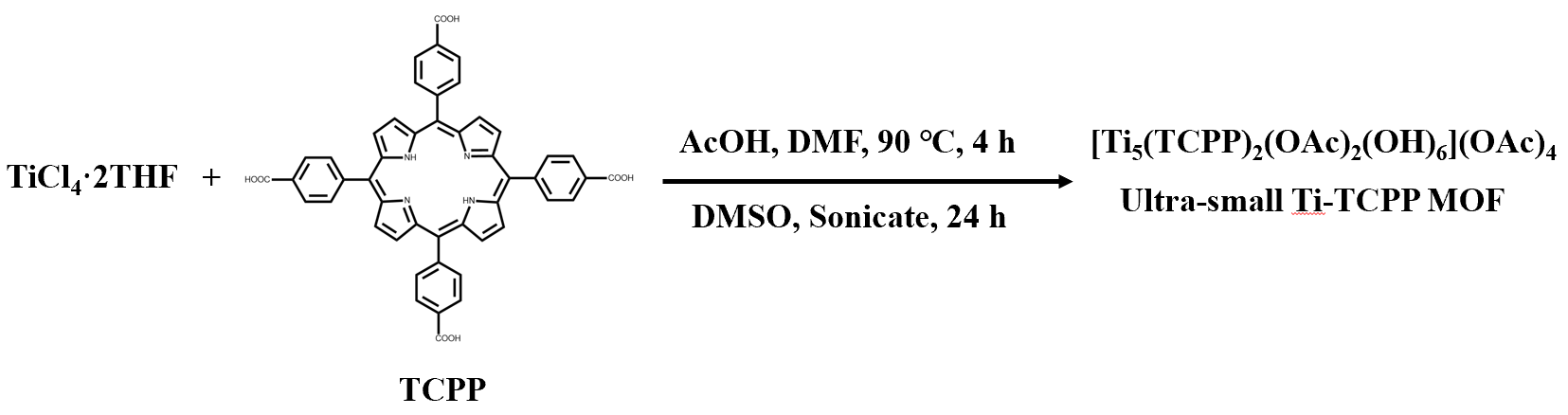


**Fig. S1** Structural formula and synthesis method of ultra-small Ti-TCPP MOF.


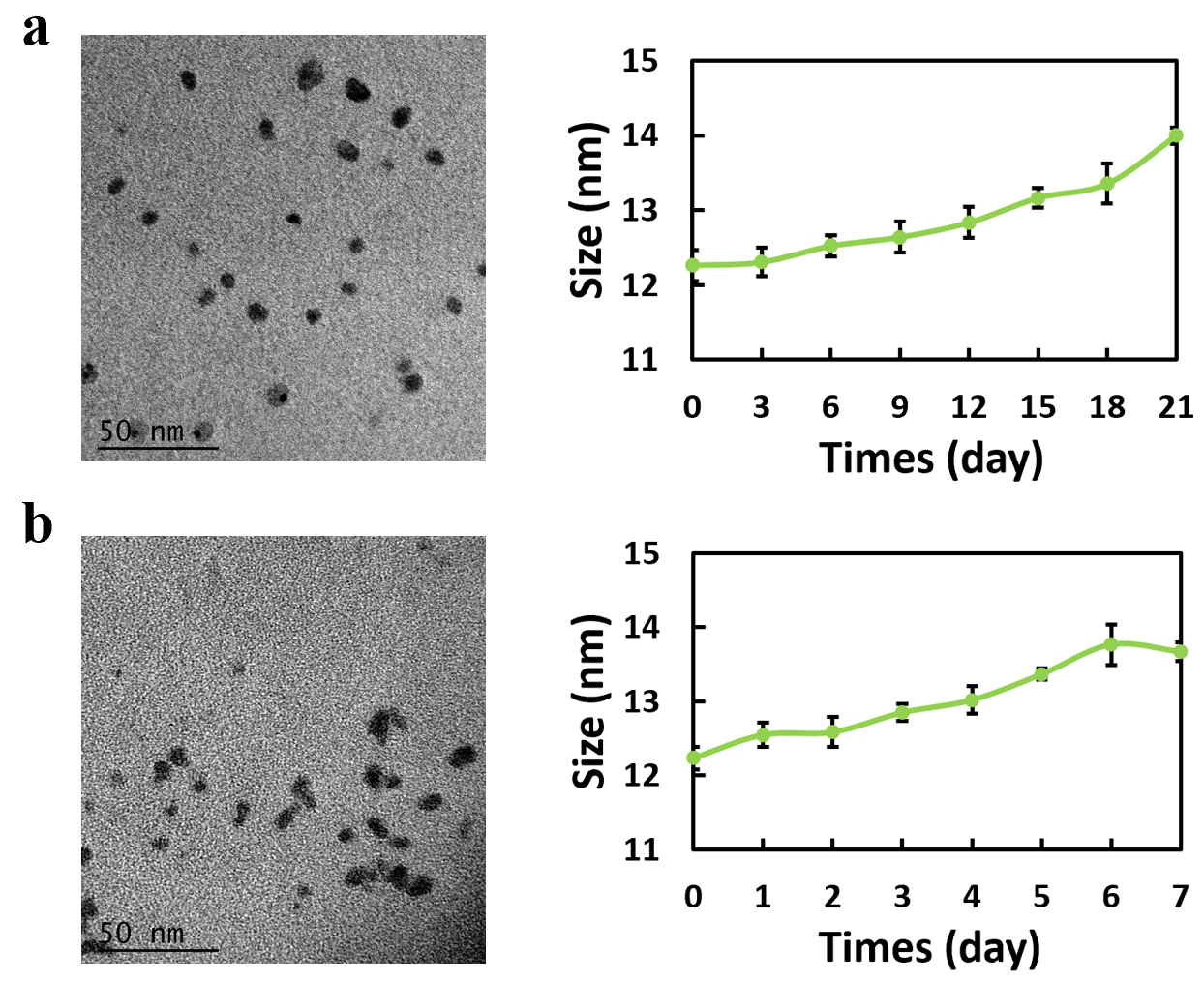


**Fig. S2** TEM images and DLS measurement of Ti-TCPP MOF in PBS for 21 days (a) and FBS for 7 days (b) at 4 ℃. Scale bar: 50 nm.


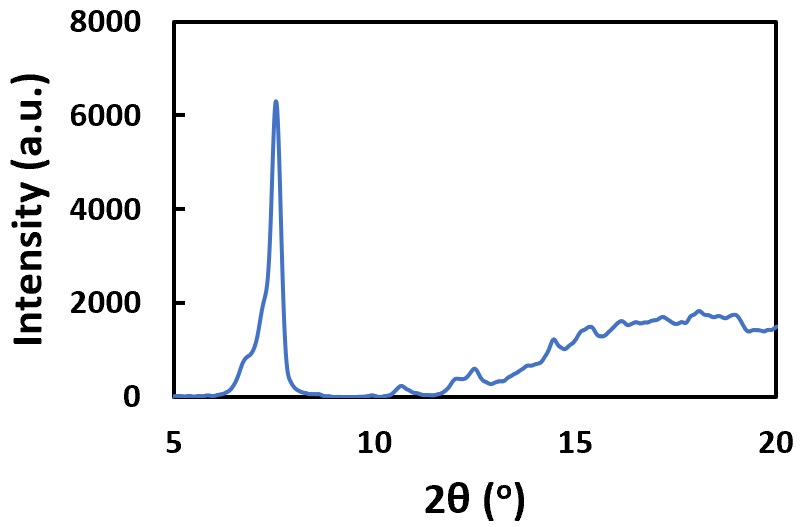


**Fig. S3** XRD pattern of Ti-TCPP MOF in PBS for 21 days at 4℃.


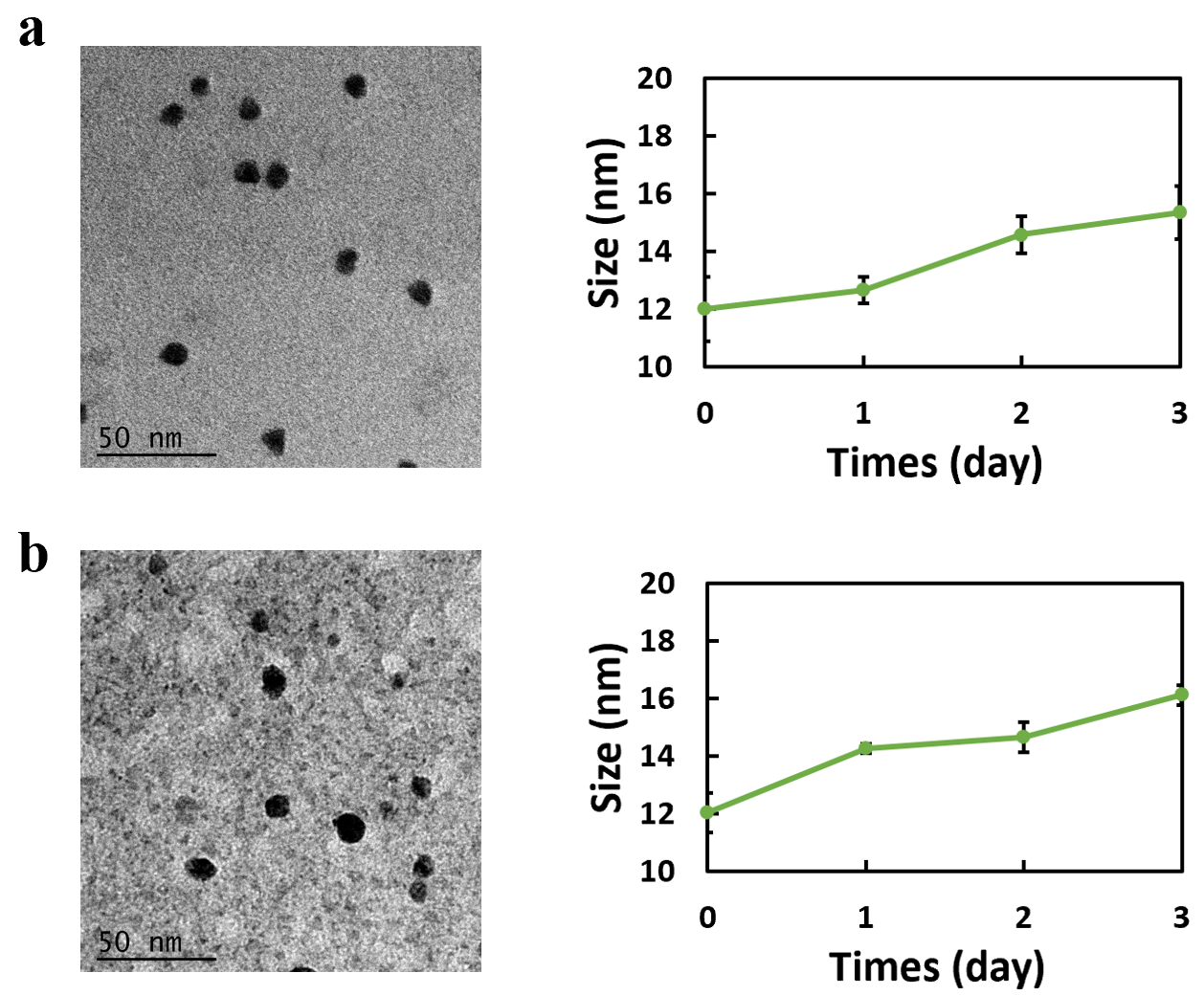


**Fig. S4** TEM images and DLS measurement of Ti-TCPP MOF in PBS (a) and FBS for 3 days (b) at 37 ℃. Scale bar: 50 nm.


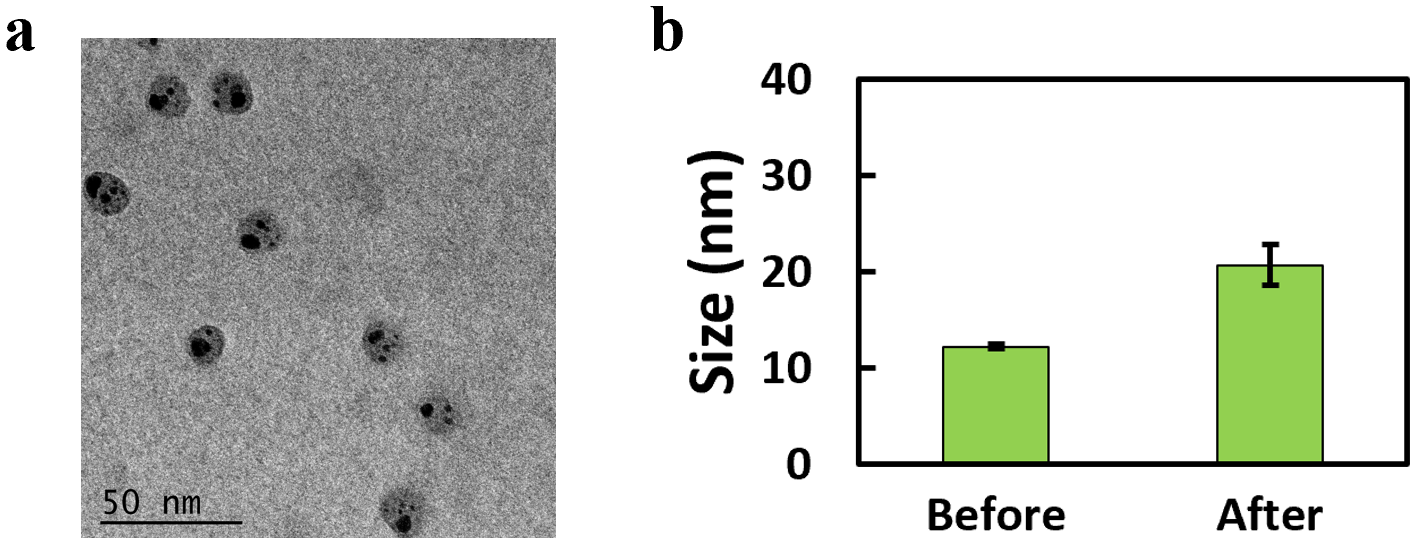


**Fig. S5** TEM images and DLS measurement of Ti-TCPP MOF in PBS after US irradiation (0.5 W/cm^2^, 1 MHz, 50% duty cycle, 1 min). Scale bar: 50 nm.


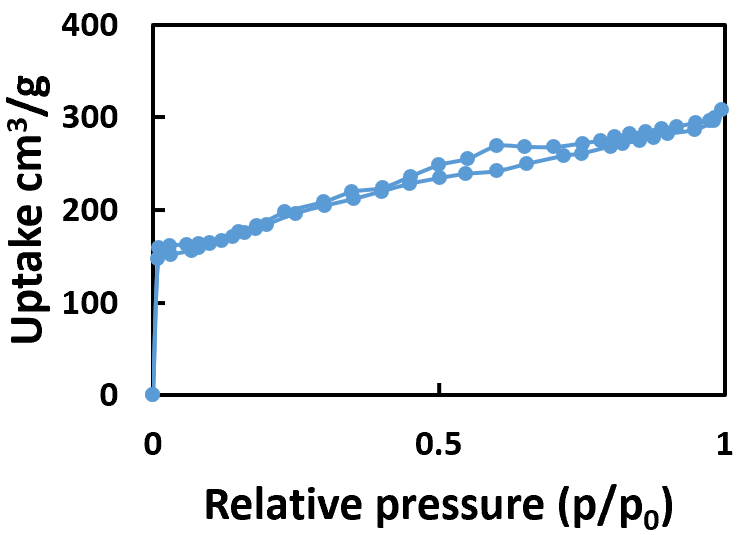


**Fig. S6** N_2_ adsorption/desorption result of ultra-small Ti-TCPP MOF.


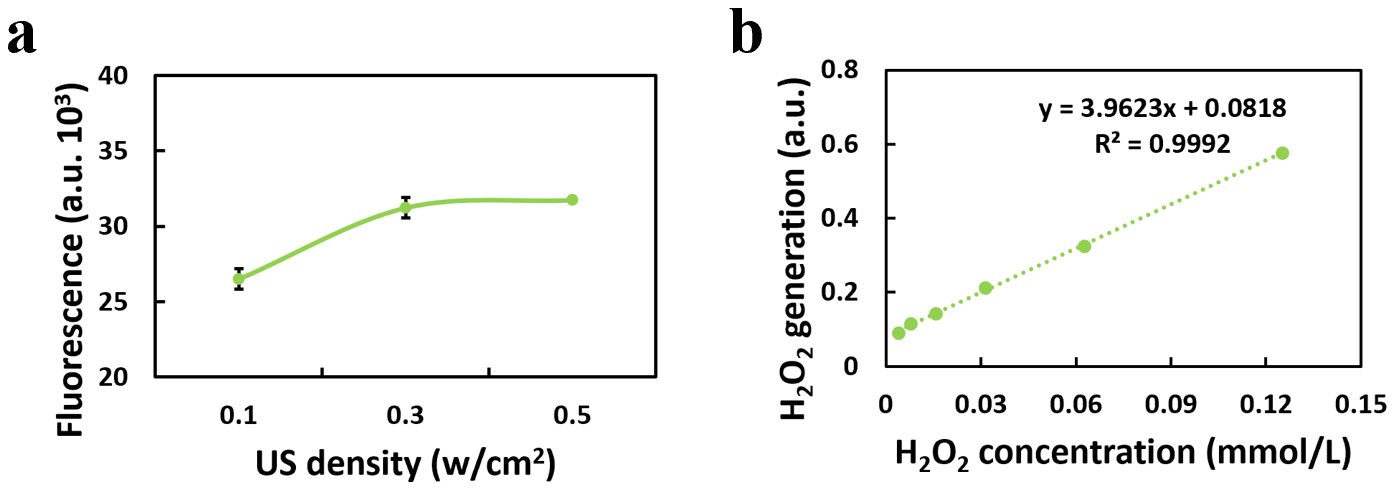


**Fig. S7** (a) Power-dependent ^1^O_2_ generation after US irradiation under normoxia conditions. (b) A standard curve of H_2_O_2_ generation measured by multiscan spectrum.


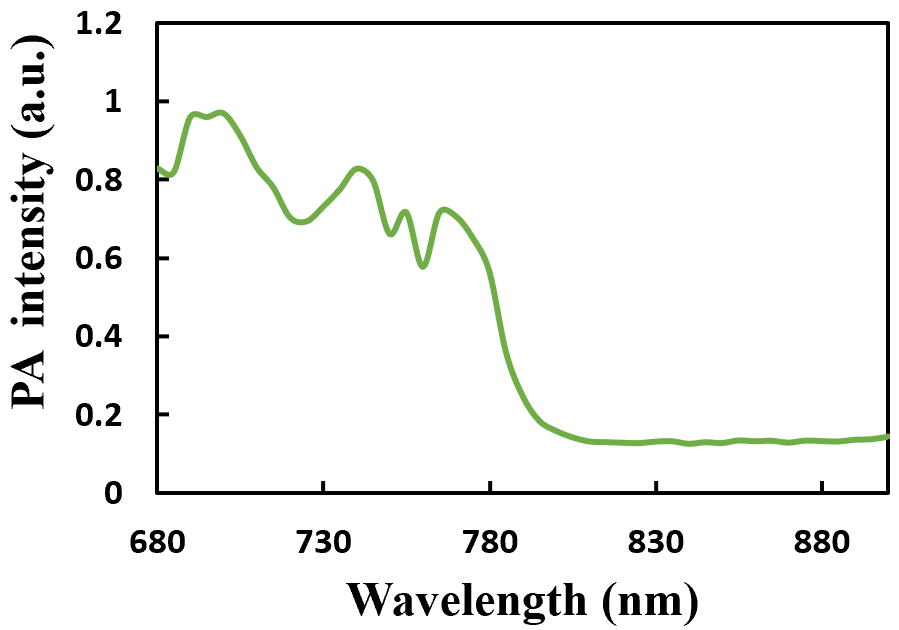


**Fig. S8** The PA signal of Ti-TCPP MOF (1 mg/mL) under 680-900 nm pulse laser irradiation *in vitro*.


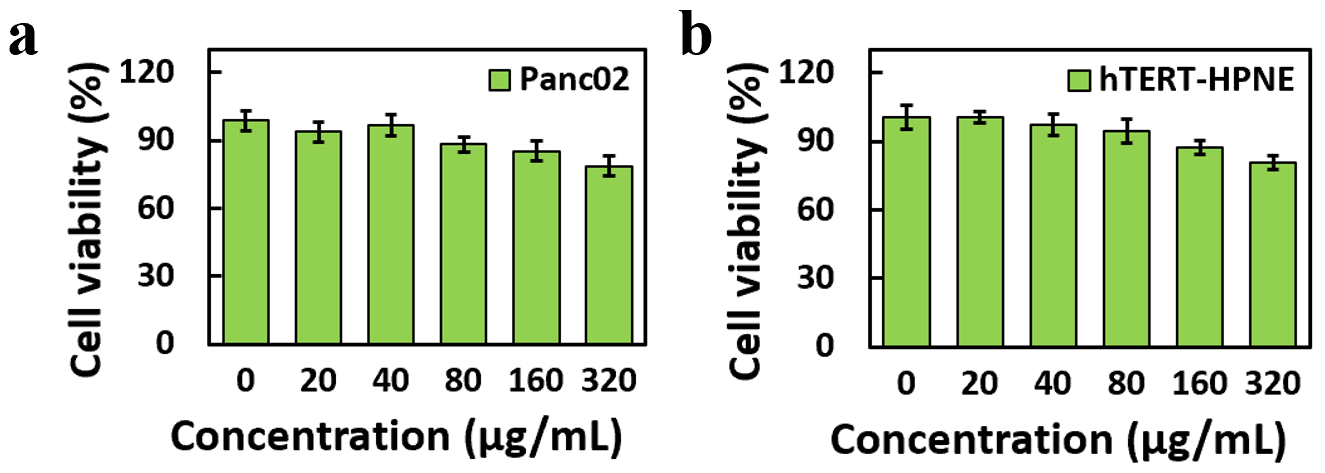


**Fig. S9** Cell viability of Panc02 (a) and hTERT-HPNE (b) cells after incubated with Ti-TCPP MOF for 24 h.


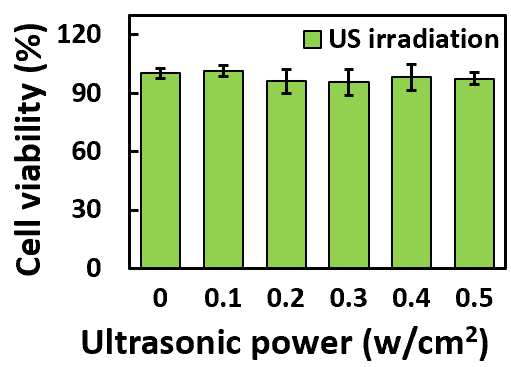


**Fig. S10** Cell viability of BxPC-3 cells after US irradiation (1 MHz, 50% duty cycle, 1 min) for 24 h.


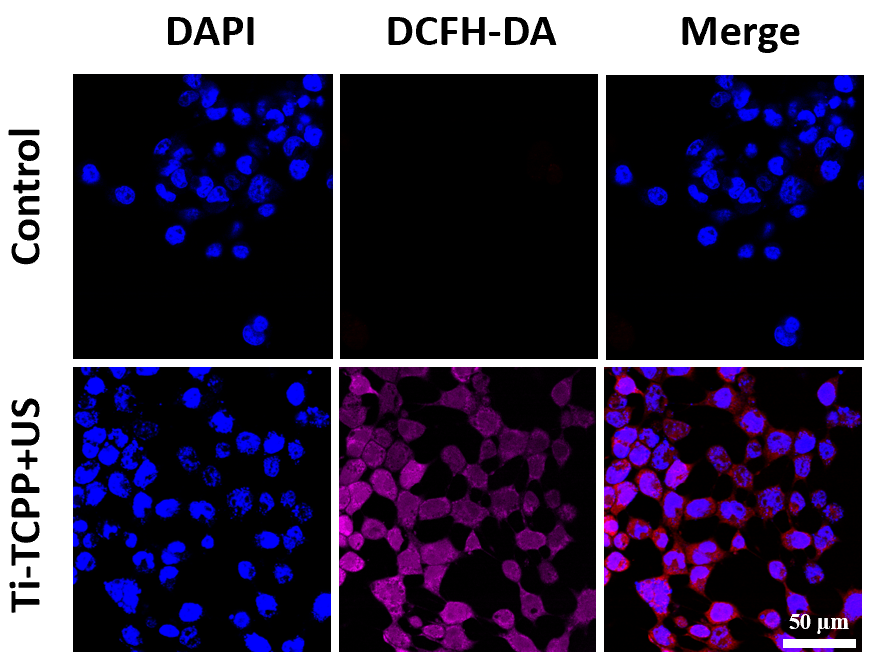


**Fig. S11** Detection of ROS generation by DCFH-DA kit in tumor cells.


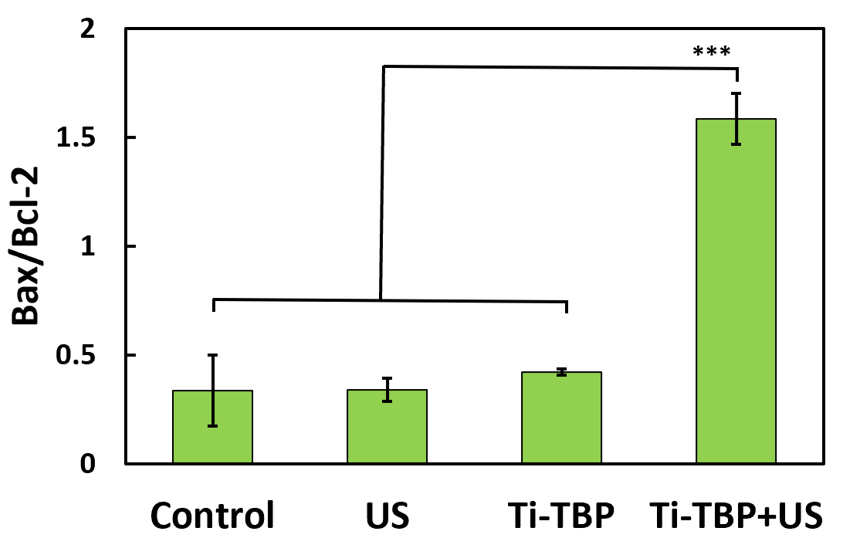


**Fig. S12** The quantified levels of Bax/Bcl-2 was analyzed by Image J software. The data were expressed as mean ± S.D. (n = 3). ***P < 0.001.


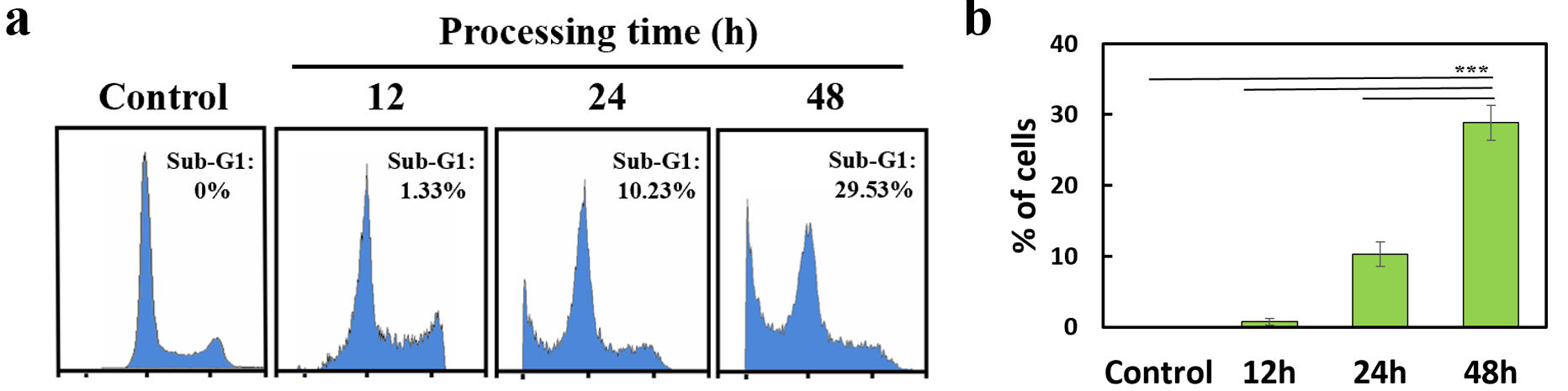


**Fig. S13** Cell cycle analysis determined by flow cytometry (a) and statistical analysis of sub-G1 phase (b) after treatment with Ti-TCPP + US at different time periods (n = 3). ***p<0.001.


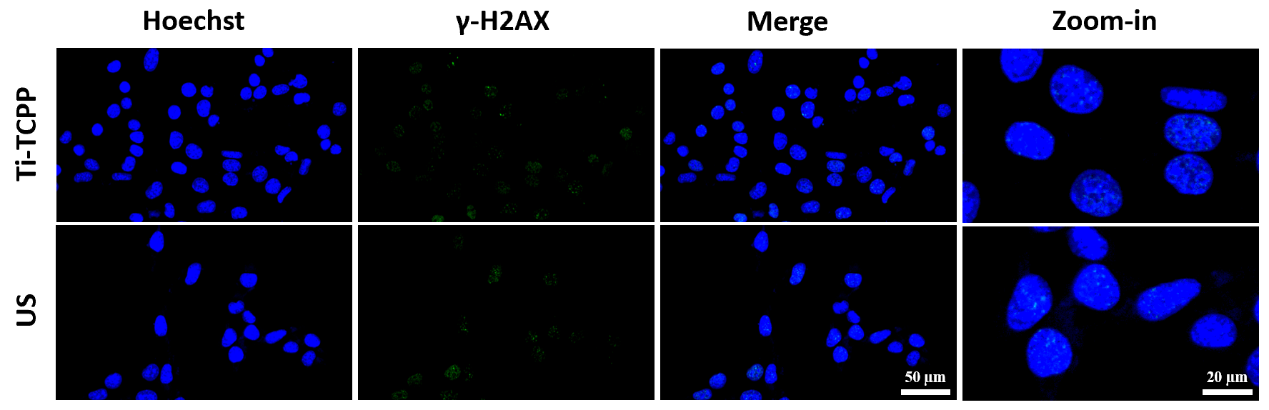


**Fig. S14** Confocal images of cancer cells in which the nuclei were stained blue with Hoechst and the γ-H2AX foci bright green following nuclear-targeting Ti-TCPP MOF treatment or US irradiation.


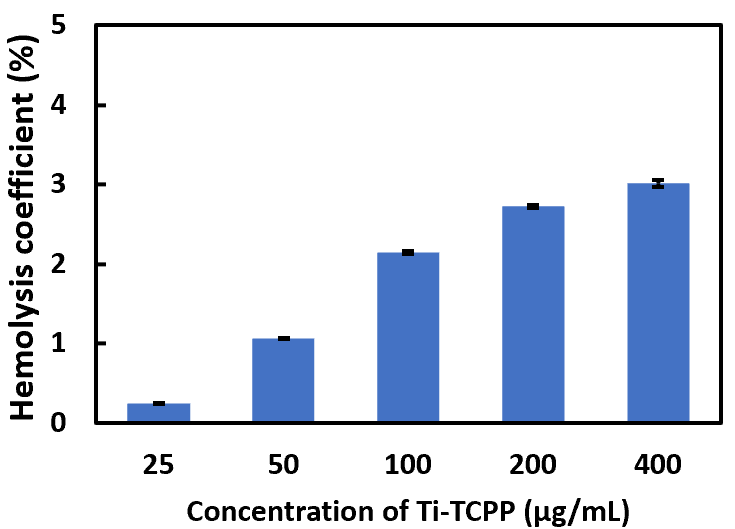


**Fig. S15** Hemolysis coefficient after incubation of RBCs with Ti-TCPP MOF (400, 200, 100, 50, or 25 µg/mL) or water, respectively (n=3). The hemolysis coefficient of Ti-TCPP MOF was less than 5%, indicating the good biocompatibility of Ti-TCPP MOF.


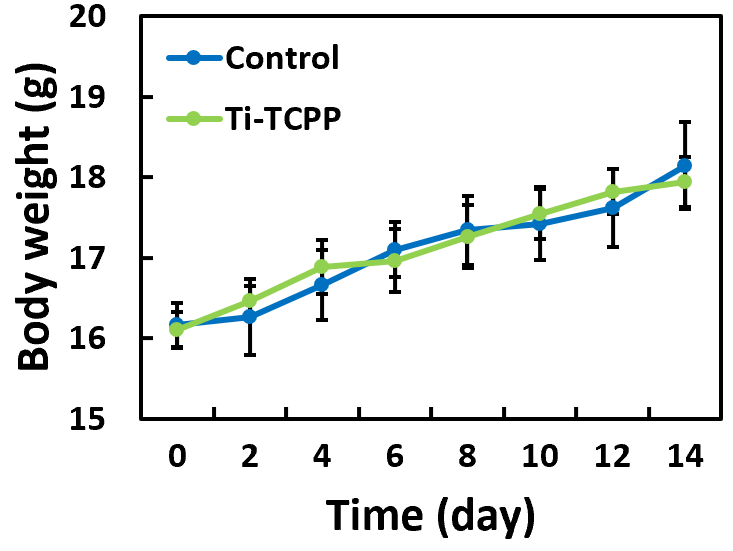


**Fig. S16** The body weight changes of healthy mice treated by PBS or Ti-TCPP MOF during 14 days.


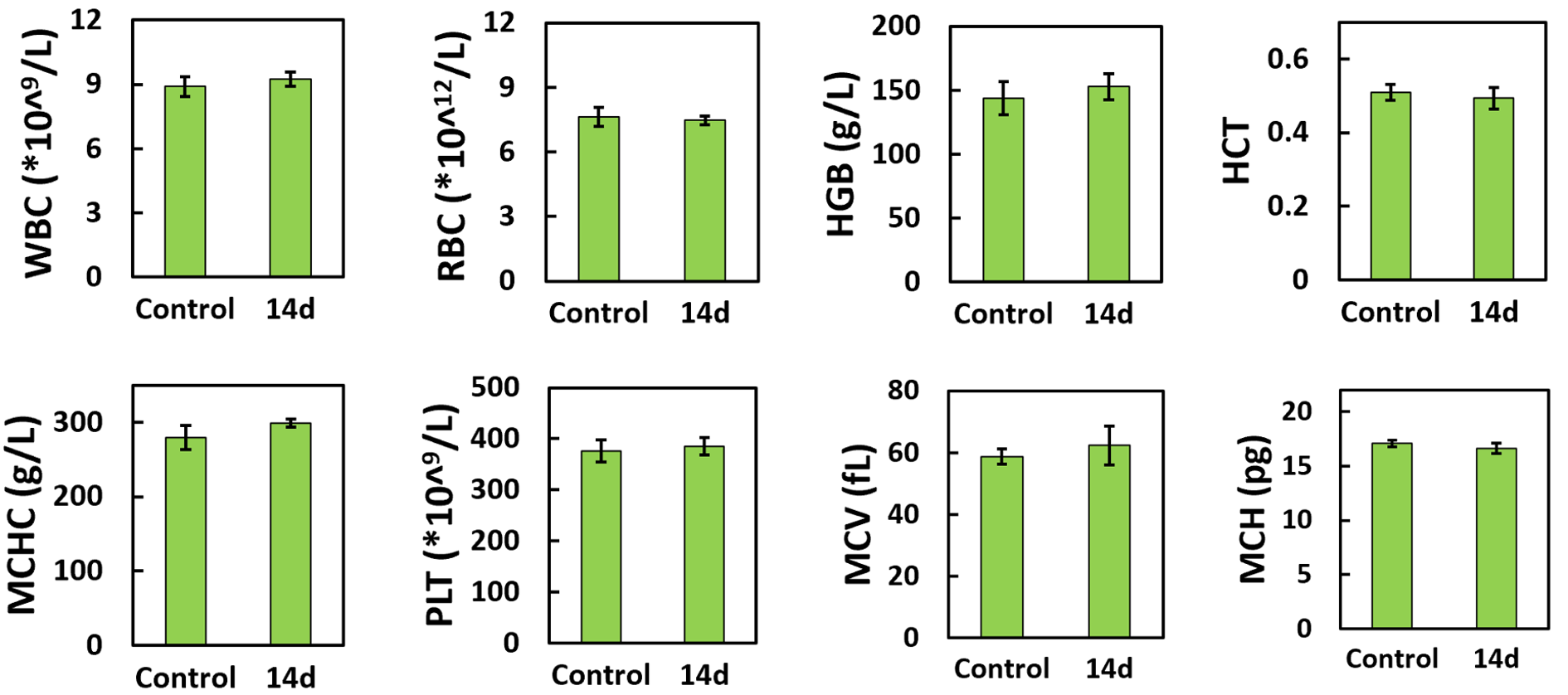


**Fig. S17** Blood routine analysis. Blood levels of WBC, RBC, HGB, HCT, MCHC, PLT, MCV and MCH of health mice after 14 d post injection (i.v.) of Ti-TCPP MOF, PBS was set as control.


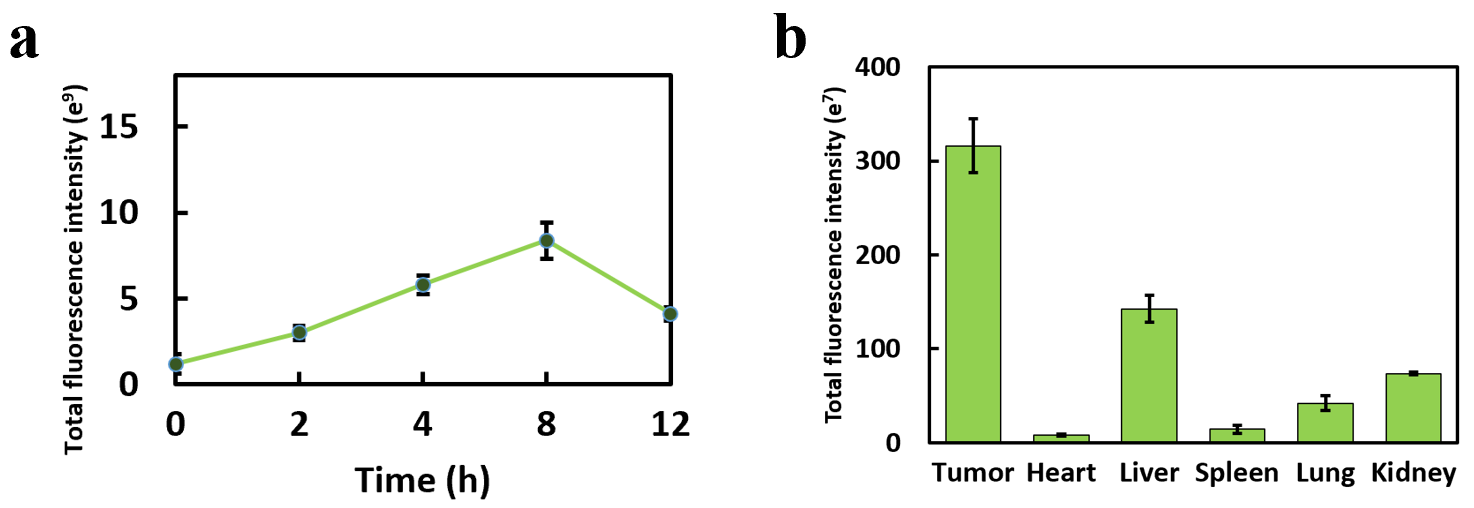


**Fig. S18** (a) Statistics analysis of tumor fluorescence intensity after i.v. Ti-TCPP MOF injection at different time period (n=3). (b) Statistics analysis of fluorescence intensity of main organs 12 h after i.v. Ti-TCPP MOF injection (n=3).


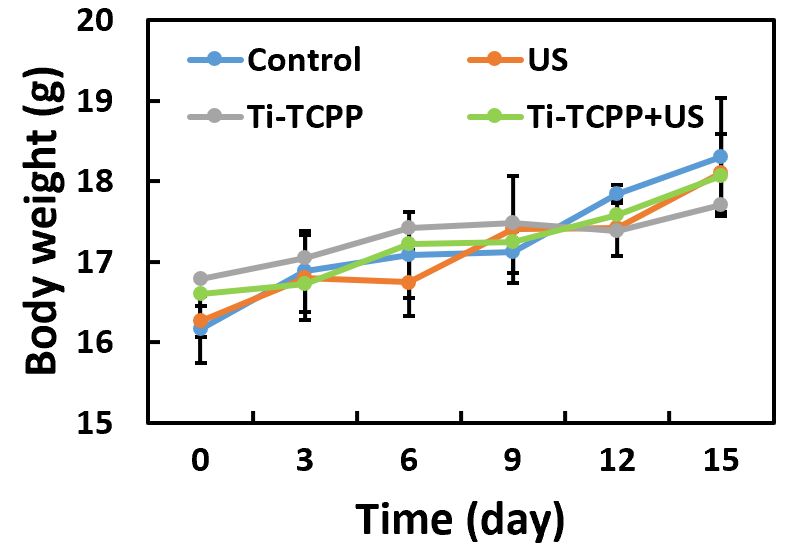


**Fig. S19** The body weight of mice after various treatment for 15 days, indicating no acute toxicity to mice major organs. n=5.


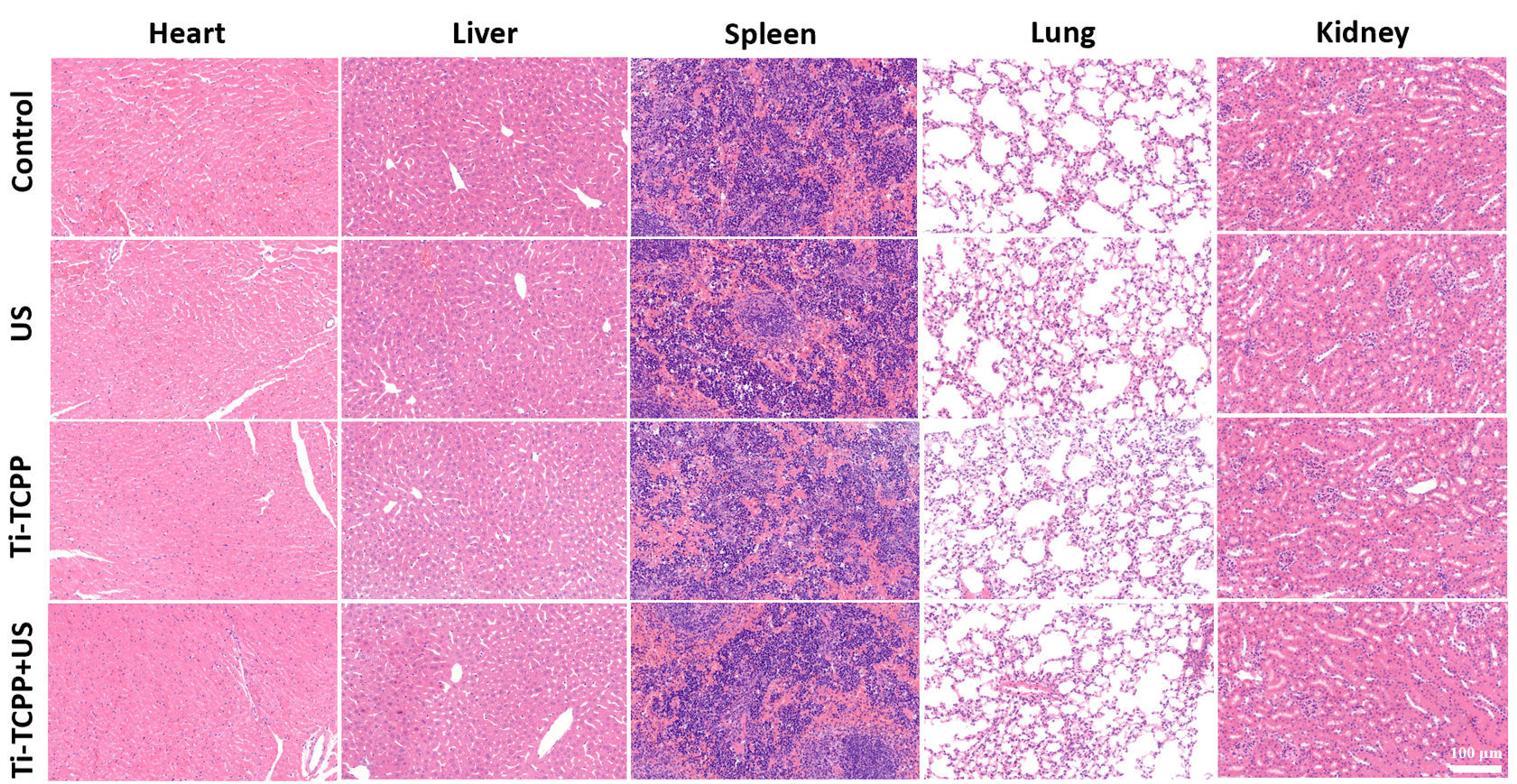


**Fig. S20** Histological analysis of the main organs (heart, liver, spleen, lung, and kidney) of un-treated mice (control) and mice treated with US, Ti-TCPP MOF and Ti-TCPP MOF+US for 15 days. Scale bar=100 µm.
